# 6-Bromoindole- and 6-Bromoindazole-Based Inhibitors of Bacterial Cystathionine γ-Lyase Containing 3-Aminothiophene-2-Carboxylate Moiety

**DOI:** 10.3390/molecules30020388

**Published:** 2025-01-17

**Authors:** Roman A. Novikov, Dmitry N. Platonov, Alexander Yu. Belyy, Konstantin V. Potapov, Maxim A. Novikov, Yury V. Tomilov, Olga I. Kechko, Tatiana A. Seregina, Anastasia S. Zemskaya, Pavel N. Solyev, Vladimir A. Mitkevich

**Affiliations:** 1 Engelhardt Institute of Molecular Biology of the Russian Academy of Sciences, 32 Vavilov St., Moscow 119991, Russia; kospotapov@yandex.ru (K.V.P.); manovikov@ioc.ac.ru (M.A.N.); olga.kechko@gmail.com (O.I.K.); tatyana.s82@gmail.com (T.A.S.); a.zemskaia@mail.ru (A.S.Z.); mitkevich@gmail.com (V.A.M.); 2 Zelinsky Institute of Organic Chemistry of the Russian Academy of Sciences, 47 Leninsky Avenue, Moscow 119991, Russia; dmplatonov@rambler.ru (D.N.P.); duospirit@gmail.com (A.Y.B.); tom@ioc.ac.ru (Y.V.T.)

**Keywords:** 6-bromoindole, 6-bromoindazole, antibiotic potentiators

## Abstract

In recent years, a number of synthetic potentiators of antibiotics have been discovered. Their action can significantly enhance the antibacterial effect and limit the spread of antibiotic resistance through inhibition of bacterial cystathionine-γ-lyase. To expand the known set of potentiators, we developed methods for the synthesis of five new representatives of 6-bromoindole derivatives—potential inhibitors of bacterial cystathionine-γ-lyase—namely potassium 3-amino-5-((6-bromoindolyl)methyl)thiophene-2-carboxylate (**MNS2**) and its 6-bromoindazole analogs (**MNS3** and **MNS4**), along with two 6-broindazole analogs of the parent compound **NL2**. Their syntheses are based on 6-bromoindole, 6-bromoindazole and methyl 5-(bromomethyl)-3-((ethoxycarbonyl)amino)thiophene-2-carboxylate as the main building blocks, assembling the rest of the heterocyclic system on their basis at the nitrogen atom. We assessed the ability of the new inhibitors to potentiate the antimicrobial activity of gentamicin.

## 1. Introduction

Antibiotic potentiators are a class of non-antibacterial active molecules that, when combined with antibiotics, enhance their activity and block one or several biochemical pathways responsible for the spread of antimicrobial resistance (ARM) [1,2,3,4,5,6]. One such ARM pathway deals with H_2_S production in microbial cells as a protection from oxidative stress [7,8,9]. In 2021, Dr. E. Nudler’s group postulated cystathionine-γ-lyase (CGL) as the primary H_2_S-generatig enzyme in two major human pathogens*, Staphylococcus aureus* and *Pseudomonas aeruginosa*, and discovered a novel class of small molecules inhibiting bacterial CGL for antibiotic potentiation [10].

**Figure 1:**
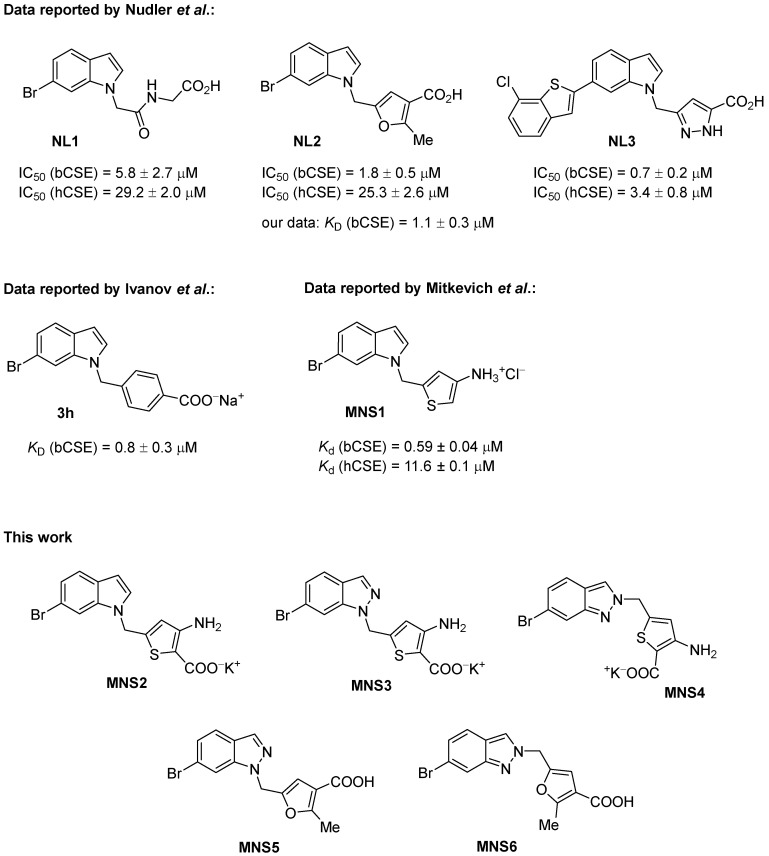
Inhibitors of bacterial cystathionine γ-lyase (CGL) for the development of antibiotic potentiators [10,11,12,13,14].

**Scheme 1:**
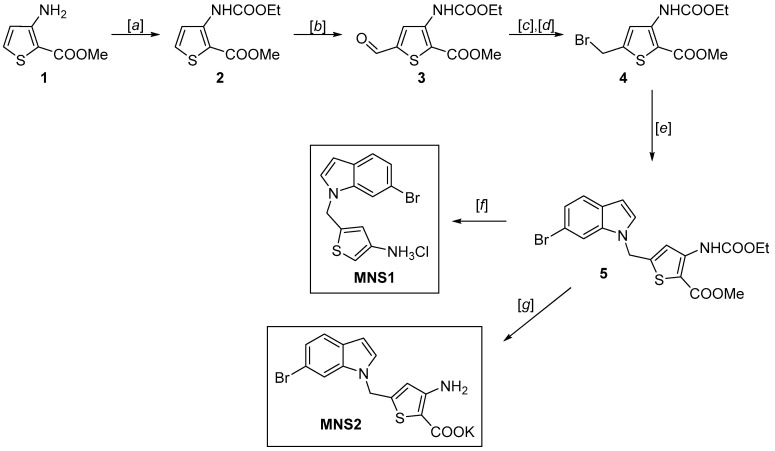
Synthesis of aminothiophenecarboxylate-based inhibitor **MNS2**. Reagents and conditions: (a) ClCO_2_Et, toluene, reflux, 10 h, 97%; (b) LDA, THF, DMF, −78 °C → −30 °C, 73%; (c) NaBH_4_, EtOH/H_2_O, 0 °C → rt, 3 h, 96%; (d) CBr_4_, PPh_3_, CH_2_Cl_2_, 0 °C → rt, 2.5 h, 80%; (e) 6-bromo-1*H*-indole, NaH, DMF, 0 °C, 24 h, 40%; (f) KOH, EtOH, THF, 60 °C, 24 h, 85%; (g) NaOH, H_2_O, reflux, then HCl, H_2_O, 89%.

The most active inhibitors of bacterial CGL are represented by the 6-bromoindole class of derivatives [10]. Since 2021, several variants of such derivatives have been synthesized with good activity at micromolar and submicromolar concentrations bearing additional furan [10,11], pyrazoline [12] or thiophene [13] heterocycles in their structure, as well as the benzoic acid moiety [14,15] (Scheme 1). The first series—**NL1**, **NL2** and **NL3** inhibitors—were introduced by Dr. E. Nudler’s group [10], and then, their synthesis was subsequently optimized for preparative procedures in gram quantities [11,12]. A number of compounds with the 6-bromoindole moiety were inspired by the **NL2** structure, including the aminothiophene derivative **MNS1** that we recently synthesized and characterized as superior to **NL2** in terms of activity toward CGL [4]. **MNS1** features one of the highest binding constants, with CGL at a level of 600 nM. Biological testing has revealed isosteric resemblance and a number of structural aspects that may give an opportunity to improve the targeted development of new inhibitors, although it has not been possible to omit the 6-bromoindole moiety so far. In this study, we focus on further modifications of **NL2**- and **MNS1**-based inhibitors, repositioning their structural moieties and further replacing the 6-bromoindole moiety with the 6-bromoindazole residue (Figure 1).

## 2. Results and Discussion

### 2.1. Synthesis of Thiophene Analog **MNS2**

The **MNS1** inhibitor is designed as a 3-aminothiophene derivative, but unlike the **NL2** inhibitor, it has no carboxylate group, maintaining high activity and binding to CGL (Figure 1). To further develop this type of structure, we synthesized and tested an **MNS1** analog titled **MNS2** with the additional carboxylate group at position 2 of the thiophene ring. The **MNS2** inhibitor is a derivative of 3-aminothiophene-2-carboxylic acid linked through the methylene bridge to the 6-bromoindole moiety (Scheme 1). The synthesis was based on the use of methyl 3-aminothiophene-2-carboxylate as the key building block, which we synthesized using the described technique from chloroacrylonitrile and ethyl thioglycolate [16,17].

Unlike **MNS1**, preserving the key carboxylate group in the final steps of **MNS2** synthesis was challenging due to the greater ease of decarboxylation under acid conditions required to unblock the protective group. To prevent this side effect, we had to change the protective group from butoxycarbonyl (BOC) to ethoxycarbonyl for amine **1**, which provides greater stability of the carboxylate group and allows milder conditions to be used. At the same time, the ethoxycarbonyl protective group, similar to BOC, has high stability toward lithium bases and reducing agents. The formed methyl 3-(ethoxycarbonyl)amino)thiophene-2-carboxylate **2** was subjected to lithiation with LDA, followed by treatment of the generated lithium derivative with DMF [18]. The reduction of aldehyde **3** with sodium borohydride and substitution of the hydroxyl group with bromide using CBr_4_ and PPh_3_ yielded methyl 5-(bromomethyl)-3-((ethoxycarbonyl)amino)thiophene-2-carboxylate **4**. The following coupling of the resulting **4** with 6-bromoindole and subsequent hydrolysis of the ester group allowed us to obtain the final product **MNS2**.

Cleavage of the ethoxycarbonyl protective group can be performed as a “one-pot” procedure along with hydrolysis of the ester group in the last step to give the target **MNS2** as a potassium salt. This method reduces the possibility of side decarboxylation. Moreover, the resulting **MNS2** salt is much more stable than the free acid, which is convenient for further studies.

### 2.2. Synthesis of 6-Bromoindazole Derivatives ***MNS3*** and ***MNS4***

**Scheme 2:**
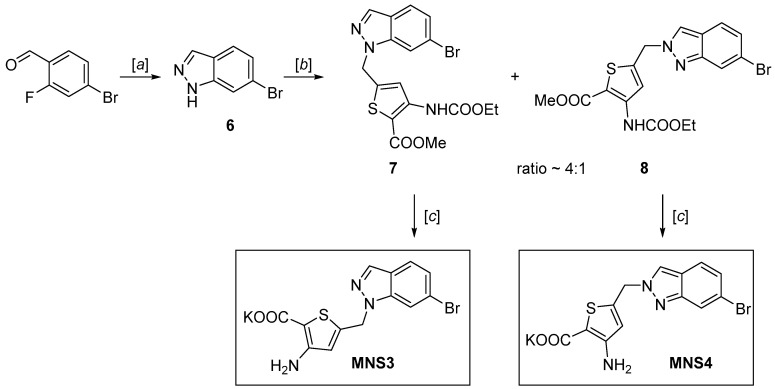
Synthesis of 6-bromoindazole analog inhibitors **MNS3** and **MNS4**. Reagents and conditions: (a) N_2_H_4_∙H_2_O, DMSO, 120 °C, 12 h, 99%; (b) NaH, DMF, 0 °C → rt, 12 h, 36% for the major isomer and 8% for the minor isomer; (c) KOH, EtOH, THF, 60 °C, 24 h, 90% for **MNS3** and 92% for **MNS4**.

The 6-bromoindazole analogs of **MNS3** and **MNS4** were synthesized using a similar procedure (Scheme 2): 6-bromoindazole was synthesized using the reaction of 4-bromo-2-fluorobenzaldehyde and hydrazine hydrate heating in DMSO [19,20], as previously described in the literature, and was alkylated with thiophene derivative **4**. The reactivity of 6-bromoindazole is comparable to that of 6-bromoindole; it undergoes nucleophilic substitution under virtually the same conditions. The coupling of 5-(bromomethyl)-3-((ethoxycarbonyl)amino)-thiophene-2-carboxylate forms two compounds in a 4:1 ratio—the products of the competing alkylation at positions 1 and 2 of the nitrogen atoms of the indazole heterocycle [21,22]. The obtained mixture of isomers was separated by silica gel column chromatography in the petroleum ether/EtOAc (4:1) system to be used as individual products. The “one-pot” hydrolysis conditions were identical to the synthesis of **MNS2**, giving **MNS3** and **MNS4** as potassium salts.

### 2.3. Synthesis of the 6-Bromoindazole Analogs of ***NL2*** (***MNS5*** and ***MNS6***)

**Scheme 3:**
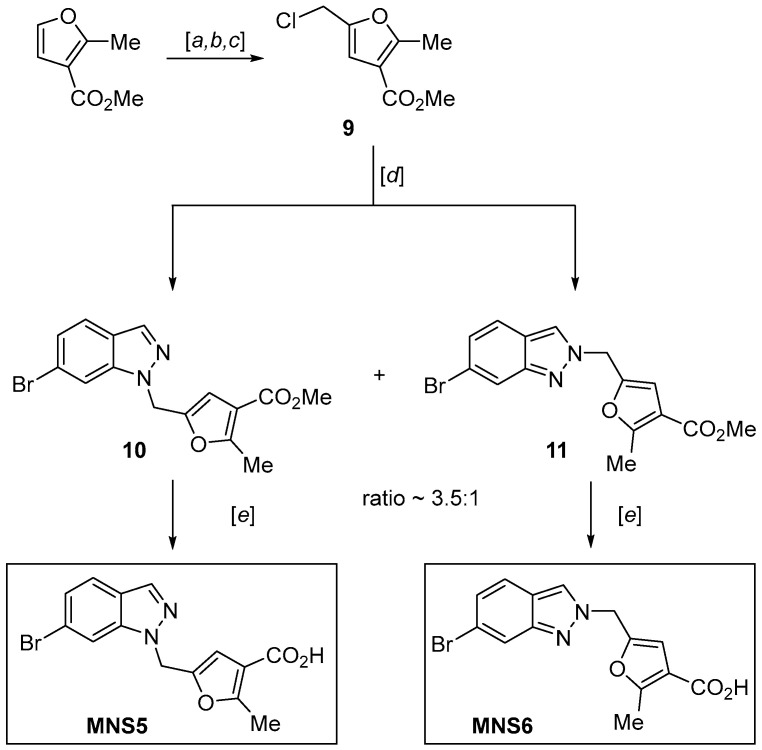
Synthesis of 6-bromoindazole inhibitors **MNS5** and **MNS6**. Reagents and conditions: (a) POCl_3_, DMF, 0 °C → 100 °C, 2 h, 73%; (b) NaBH_4_, CH_2_Cl_2_, MeOH, 0 °C → rt, 1 h, 91%; (c) MsCl, Et_3_N, CH_2_Cl_2_, 0 °C → rt, 12 h, 75%; (d) NaH, 6, DMF, 0 °C → rt, 12 h, 56% for the major isomer and 17% for the minor isomer; (e) NaOH, MeOH, H_2_O, rt, then HCl, H_2_O, 72% for **MNS5** and 71% for **MNS6**.

To evaluate the isosteric effect of the 6-bromoindazole moiety on activity, two new analogs of **NL2** were synthesized. Following the procedure for **NL2**, 6-bromoindazole-containing **MNS5** and **MNS6** were synthesized by coupling two heterocyclic fragments with the following hydrolysis of the ester group in the last step (Scheme 3). Methyl 5-(chloromethyl)-2-methylfuran-3-carboxylate **9** can be prepared from commercially available methyl 2-methylfuran-3-carboxylate via the previously reported synthetic route of formylation reactions, reduction of the resulting aldehyde with sodium borohydride and substitution of the hydroxyl group with chloride via methanesulfonyl chloride [11,23,24]. Product **9** was used as the furan building block for direct alkylation of 6-bromoindazole.

Similar to the synthesis of 6-bromoindazole derivatives **MNS3** and **MNS4**, alkylation of 6-bromoindazole with chloromethylfuran derivative **9** gave two compounds, **10** and **11,** in a 3.5:1 ratio as products of competing alkylation reactions at the N1 and N2 positions of the indazole heterocycles. Both the target product and the side product were isolated, purified and subjected to further hydrolysis in NaOH solution to give inhibitors **MNS5** and **MNS6**.

All the final products **MNS2**–**MNS6** were tested as antibiotic potentiators in bacteria.

### 2.4. Determination of the Potentiating Activity in Bacillus subtilis Cells

**Figure 2:**
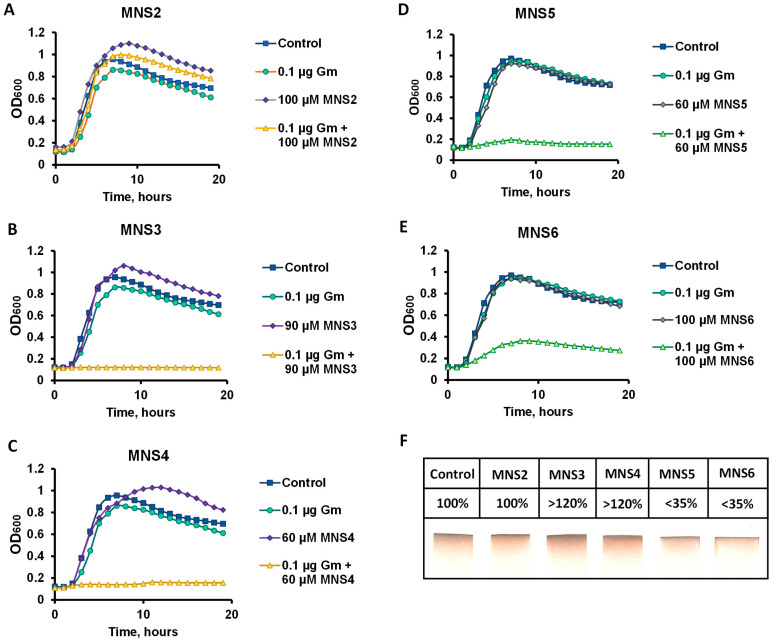
Determination of the potentiating properties of compounds. Representative growth curves of *B. subtilis* 168 strain in the presence of 0.1 μg/mL gentamicin (Gm) and potentiators: **MNS2** (**A**), **MNS3** (**B**), **MNS4** (**C**), **MNS5** (**D**) and **MNS6** (**E**). The cells were grown at 37 °C with aeration in a Bioscreen C automated growth assay system. The curves represent the mean values of three independent experiments with an error of <5%. Evaluation of hydrogen sulfide production by *B. subtilis* 168 cells in the presence of the potentiators studied (**F**): **MNS2** (100 µM), **MNS3** (90 µM), **MNS4** (60 µM), **MNS5** (60 µM) and **MNS6** (100 µM). The filter paper strips soaked in 2% lead acetate solution show brown staining with PbS salt as a result of H_2_S evaporation by the bacterial suspension. The numbers indicate H_2_S production relative to wild-type cells.

We selected the *B. subtilis* 168 strain as the model micro-organism to determine the potentiating properties of the new compounds. The active center of the proposed target protein, cystathionine γ-lyase, has a conservative structure for a wide range of micro-organisms, including pathogens; thus, *B. subtilis* can be used as a less toxic model object containing the CGL enzyme for testing [9]. The efficiency of CGL inhibitors was assessed by the standard minimum inhibitory concentration (MIC) assay. The potentiating properties were investigated according to the technique reported previously for **NL2 [10]**. The MIC of gentamicin for *B. subtilis* cells is 1 μg/mL, which is consistent with known CLSI breakpoints for the genus *Bacillus* [25]. Of all the derivatives obtained, compounds **MNS3** and **MNS4** (Figure 2B,C) at concentrations of 90 and 60 µM, respectively, exhibited the highest inhibitory activity in the presence of 0.1 MIC of gentamicin. The **MNS5** inhibitor completely suppressed cell growth at a concentration of 60 µM, which is comparable to the results for **MNS4** (Figure 2C). However, **MNS2** failed to inhibit *B. subtilis* growth in combinations with gentamicin in the concentration range from 50 to 100 µM. All the inhibitors studied did not exert their own toxic effect on the growth of *B. subtilis* cells (Figure 2A–E). In addition, we tested the effect of the compounds on the generation of hydrogen sulfide by the *B. subtilis* cell culture (Figure 2F). It was revealed that the furan-containing products **MNS5** and **MNS6** significantly reduced the rate of hydrogen sulfide generation compared to the control group of cells, while, in contrast, thiophene-containing **MNS2** and **MNS3** enhanced the production of H_2_S. The latter effect, however, may be attributed to the presence of the sulfur atom in the structure of these inhibitors assuming their possible hydrolysis if not bound to the enzyme in the cellular environment.

**Figure 3:**
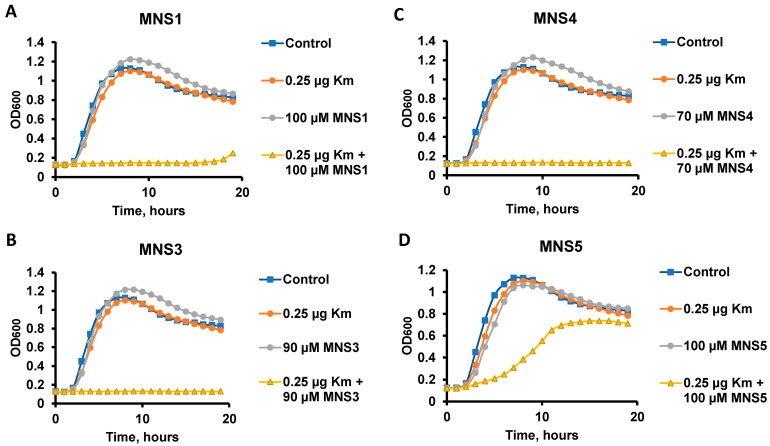
Determination of the potentiating properties of compounds. Representative growth curves of *B. subtilis* 168 strain in the presence of 0.25 μg/mL kanamycin A (Km) and potentiators: **MNS2** (**A**), **MNS3** (**B**), **MNS4** (**C**), **MNS5** (**D**).

In addition, **MNS3**, **MNS4** and **MNS5** were selected as the most active compounds for testing their potentiating properties with kanamycin A (Km). The potentiating properties and minimum inhibitory concentration (MIC) were assessed similarly to the testing with gentamicin, and additionally, the previously reported **MNS1** was used for comparison (Figure 3). The MIC of kanamycin A for *B. subtilis* cells is 2 μg/mL. Of all the derivatives obtained, compounds **MNS3** and **MNS4** (Figure 3B,C) at concentrations of 90 and 70 µM, respectively, exhibited the highest inhibitory activity in the presence of 0.1 MIC of kanamycin. These results were superior to the parent **MNS1** (both for the currently studied kanamycin A potentiation and for previously known data for gentamicin potentiation) and the new **MNS5**. A lower cytotoxic effect on HEK273T culture (Table 1) allows us to conclude that MNS3 is the hit compound in the series (the experimental curves for CC_50_ estimation via the MTT test are given in the Supplementary Information File).

## 3. Materials and Methods

### 3.1. General Experimental Details

All reagents and catalysts were purchased from Sigma-Aldrich (St. Louis, MO, USA), Acros (Belgium), J&K Scientific (USA, San Jose, CA, USA) and TCI Europe (Zwijndrecht, Belgium) and used without further purification unless otherwise mentioned. TLC analysis was performed on Silufol chromatographic plates. For preparative chromatography, silica gel 60 (0.040–0.063 mm) was used. ^1^H, ^13^C NMR spectra were recorded on a Bruker AVANCE II 300 MHz (300.1 and 75.5 MHz, respectively) and a Bruker AMX III 400 MHz (400.1 and 100.6 MHz, respectively), QOne Quantum-I-Plus AS (600 and 151 MHz, respectively) spectrometers in CDCl_3_ containing 0.05% Me_4_Si as the internal standard. HRMS spectra were registered on a Bruker Daltonics micrOTOF-Q II device with electrospray ionization in positive ion mode. Samples were loaded using an autosampler from acetonitrile solution in an Agilent 1260 liquid chromatograph equipped with an Agilent Poroshell 120 EC-C18 column (3.0 × 50 mm; 2.7 μm). The flow rate was 0.4 mL min^−1^, and elution was performed at the following gradient of CH_3_CN (A) in H_2_O: 0–6 min—0–85% A, 6–7.5 min—85% A, 7.5–8 min—85–0% A, 8–10 min—0% A. The eluent flow was injected into the spray chamber of the mass spectrometer as a detector.

### 3.2. Methyl 3-((ethoxycarbonyl)amino)thiophene-2-carboxylate (2)

Ethyl chloroformate (1.38 g, 12.7 mmol) was added to a solution of methyl 3-aminothiophene-2-carboxylate (2.00 g, 12.7 mmol) in 10 mL of toluene and refluxed for 10 h. The resulting solution was cooled to rt and evaporated. The crude product was purified via crystallization from methanol (2.80 g, 97%). Mp = 64–65 °C. The NMR data and Mp are in full agreement with the previously published data [26].

^1^H NMR (300 MHz, CDCl_3_) δ = 9.51 (s, 1H, N*H*), 7.91 (d, *J* = 5.5, 1H, C*H*(Ar)), 7.46 (d, *J* = 5.5, 1H, C*H*(Ar)), 4.26 (q, *J* = 7.1, 2H, OC*H*_2_), 3.90 (s, 3H, OC*H*_3_), 1.34 (t, *J* = 7.1, 3H, C*H*_3_).

### 3.3. Methyl 3-((ethoxycarbonyl)amino)-5-formylthiophene-2-carboxylate (3)

A 2.5 M solution of BuLi in n-hexane (42 mL, 104 mmol) was added drop-wise to a solution of diisopropylamine (11.90 g, 117 mmol) in 50 mL of anhydrous THF under argon atmosphere, with temperature control of the mixture not exceeding −50 °C. The resulting solution was pre-cooled to −78 °C, and a solution of methyl 3-((ethoxycarbonyl)amino)thiophene-2-carboxylate (**2**) (8.40 g, 32.7 mmol) in 30 mL of THF was added. The reaction mixture was stirred for 45 min at −78 °C; then, anhydrous DMF (12.0 g, 118 mmol) was added, maintaining the solution temperature below −65 °C. The mixture was kept at −78 °C for another 15 min, then heated to −30 °C and treated with 6M aqueous hydrochloric acid (36.8 mL, 221 mmol). After the temperature had stabilized, water was added to the resulting suspension until LiCl was completely dissolved. The organic phase was separated, washed with a saturated NaCl solution (3 × 50 mL), dried over Na_2_SO_4_ and evaporated in vacuo. The product was purified via crystallization from methanol to render light yellow crystals (6.81 g, 73%).

^1^H NMR (300 MHz, CDCl_3_) δ = 9.98 (s, 1H, C*H*O), 9.43 (s, 1H, N*H*), 8.56 (s, 1H, C*H*(Ar)), 4.29 (q, *J* = 7.1, 2H, OC*H*_2_), 3.95 (s, 3H, OCH_3_), 1.36 (t, *J* = 7.1, 3H, CH_3_). ^13^C NMR (75 MHz, CDCl_3_) δ = 183.9, 164.2, 152.9, 145.3, 144.4, 128.4, 115.1, 62.0, 52.5, 14.4. HRMS (ESI) of C_10_H_11_NO_5_S, *m/z*: calcd for [M+H]^+^ 258.0431; found 258.0438.

### 3.4. Methyl 5-(bromomethyl)-3-((ethoxycarbonyl)amino)thiophene-2-carboxylate (4)

NaBH_4_ (0.62 g, 16.3 mmol) was added to a suspension of methyl 3-((ethoxycarbonyl)amino)-5-formylthiophene-2-carboxylate (**3**) (9.67 g, 33.9 mmol) in a mixture of 250 mL of ethanol and 250 mL of water at 0 °C. The mixture was stirred for 1 h at 0 °C and 1 h at room temperature. Then, a second portion of NaBH_4_ (0.62 g, 16.3 mmol) was added in two steps over 2 h. The resulting suspension was stirred at room temperature for 1 h and then diluted with 500 mL of water. The mixture was extracted with ethyl acetate (2 × 250 mL), and the organic layers were combined, dried over MgSO_4_ and evaporated in vacuo. The product was used in further reactions without purification.

CBr_4_ (8.19 g, 24.6 mmol) was added portion-wise to a solution of dry residue obtained in the previous stage and triphenylphosphine (6.47 g, 24.6 mmol) in 50 mL of CH_2_Cl_2_ at 0 °C. The reaction mixture was stirred for 30 min at 0 °C and 2 h at room temperature and then evaporated to dryness. The product was extracted from the solid residue with a mixture of Et_2_O and hexane (1:1). The organic salts were combined, passed through a pad of silica gel and evaporated in vacuo. The product was obtained as a white powder (5.74 g, 80%) and used in further transformations without purification. Mp = 100–101 °C.

^1^H NMR (300 MHz, CDCl_3_) δ = 9.43 (s, 1H, N*H*), 7.94 (s, 1H, C*H*(Ar)), 4.61 (s, 2H, C*H*_2_), 4.24 (q, *J* = 7.1, 2H, OC*H*_2_), 3.87 (s, 3H, OCH_3_), 1.33 (t, *J* = 7.1, C*H*_3_). ^13^C NMR (75 MHz, CDCl_3_) δ = 183.7, 164.2, 152.9, 145.3, 144.4, 128.4, 115.1, 62.0, 52.5, 14.4. HRMS (ESI) of C_10_H_12_BrNO_4_S, *m/z*: calcd for [M+H]^+^ 319.9587 and 321.9566; found 319.9595 and 321.9572.

### 3.5. Methyl 5-((6-bromo-1H-indol-1-yl)methyl)-3-((ethoxycarbonyl)amino)thiophene-2-carboxylate (5)

NaH (204 mg, 60% dispersion in oil, 5.1 mmol) was added to a solution of 6-bromoindole (1.0 g, 5.1 mmol) in 18 mL of anhydrous DMF at 0 °C. The mixture was stirred at room temperature until hydrogen release stopped (2 h). A solution of methyl 5-(bromomethyl)-3-((ethoxycarbonyl)amino)thiophene-2-carboxylate (**4**) (1.49 g, 4.6 mmol) in 2 mL of anhydrous DMF was added to the resulting solution. The reaction mixture was kept at 0 °C for 24 h; then, it was diluted with 100 mL of water. The mixture was extracted with EtOAc (3 × 50 mL), and the organic layers were combined, washed with water (3 × 50 mL) and with saturated NaCl solution (3 × 50 mL), dried over Na_2_SO_4_ and evaporated in vacuo. The substance was purified via column chromatography on silica gel using toluene as an eluent (Rf = 0.35). The product was isolated as a yellowish oil (891 mg, 40%).

^1^H NMR (300 MHz, CDCl_3_) δ 9.45 (s, 1H), 7.84 (s, 1H), 7.57−7.42 (m, 2H), 7.35−7.18 (m, 1H), 7.12 (d, *J* = 3.0 Hz, 1H), 6.55 (d, *J* = 3.0 Hz, 1H), 5.37 (s, 2H), 4.24 (q, *J* = 7.1 Hz, 2H), 3.81 (s, 3H), 1.33 (t, *J* = 7.1 Hz, 3H). ^13^C NMR (75 MHz, CDCl_3_) δ 164.2, 153.0, 146.5, 144.8, 136.7, 128.3, 127.7, 123.3, 122.4, 119.6, 115.8, 112.3, 107.9, 103.0, 61.8, 51.8, 45.7, 14.4. HRMS (ESI) of C_18_H_17_BrN_2_O_4_S, *m/z*: calcd for [M+Na]^+^ 458.9985 and 460.9965; found 458.9992 and 460.9973.

### 3.6. 6-Bromo-1H-indazol (6)

Hydrazine hydrate (2 mL) was added to a solution of 4-bromo-2-fluorobenzaldehyde (2.00 g, 9.85 mmol) in 4 mL of DMSO. The mixture was heated to 120 °C and stirred vigorously for 8 h. The reaction mixture was cooled to room temperature and left to crystallize overnight. The precipitated crystals were filtered, washed with 2 M HCl solution and water and then dried in vacuo. The product was isolated as colorless crystals (1.91 g, 99%). Mp 182–183 °. The NMR data and Mp are in full agreement with the previously published data [19,27].

^1^H NMR (300 MHz, DMSO-d_6_) δ 13.17 (s, 1H, N*H*), 8.10 (s, 1H, C*H*(Ar)), 7.87−7.48 (m, 2H, 2 × C*H*(Ar)), 7.23 (dd, *J* = 8.6, 1.6 Hz, 1H, C*H*(Ar)). ^13^C NMR (DMSO-d_6_) δ 112.2, 118.9, 121.3, 121.9, 122.9, 133.3, 140.1.

### 3.7. Methyl 5-((6-bromo-1H-indazol-1-yl)methyl)-3-((ethoxycarbonyl)amino)thiophene-2-carboxylate (7) and Methyl 5-((6-bromo-2H-indazol-2-yl)methyl)-3-((ethoxycarbonyl)amino)thiophene-2-carboxylate (8)

NaH (204 mg, 5.1 mmol, 60% dispersion in mineral oil) was added to a solution of 6-bromoindazole (1 g, 5.1 mmol) in 18 mL of DMF at 0 °C, and the mixture was stirred until hydrogen release stopped. A solution of methyl 5-(bromomethyl)-3-((ethoxycarbonyl)amino)thiophene-2-carboxylate (**4**) (1.49 g, 4.6 mmol) in 2 mL of DMF was added to the resulting mixture. The reaction mixture was kept at 0 °C for 24 h, then diluted with 100 mL of water and extracted with 3 × 50 mL of EtOAc. The combined organic layers were washed with 3 × 50 mL of water and with 3 × 50 mL of saturated NaCl solution, dried over Na_2_SO_4_ and evaporated in vacuo. The products were isolated via column chromatography on silica gel using petroleum ether/EtOAc 4:1 as an eluent (Rf = 0.25 and 0.2 for **7** and **8**). Two products were isolated as yellow oils (806 mg, yield 36% and 185 mg, yield 8%).

Major (**7**). Mp = 116–118 °C. ^1^H NMR (300 MHz, CDCl_3_) δ = 9.43 (s, 1H, N*H*), 8.03 (s, 1H, C*H*(Ar)), 7.88 (s, 1H, C*H*(Ar)), 7.66−7.53 (m, 2H, 2 × C*H*(Ar)), 7.33−7.23 (m, 1H, C*H*(Ar)), 5.66 (s, 2H, C*H*_2_), 4.24 (q, *J* = 7.1 Hz, 2H, OC*H*_2_), 3.81 (s, 3H), 1.31 (t, *J* = 7.0 Hz, 3H). ^13^C NMR (75 MHz, CDCl_3_) δ 164.2, 152.9, 145.2, 144.7, 140.1, 134.4, 124.8, 123.3, 122.4, 121.3, 120.1, 111.8, 108.2, 61.7, 51.8, 48.3, 14.4. HRMS (ESI) of C_17_H_16_BrN_3_O_4_S, *m/z*: calcd for [M+H]^+^ 319.9587 and 321.9566; found 319.9595 and 321.9572.

Minor (**8**). Mp = 137−139 °C. ^1^H NMR (300 MHz, CDCl_3_) δ 9.44 (s, 1H, N*H*), 7.94 (m, 3H, 3×C*H*(Ar)), 7.51 (m, *J* = 8.9 Hz, 1H, C*H*(Ar)), 7.17 (dd, *J* = 8.9, 1.4 Hz, 1H, C*H*(Ar)), 5.66 (s, 2H, C*H*_2_), 4.24 (q, *J* = 7.1 Hz, 2H, OC*H*_2_), 3.82 (s, 3H, OC*H*_3_), 1.31 (t, *J* = 7.1 Hz, 3H, CH_3_). ^13^C NMR (75 MHz, CDCl_3_) δ 164.1, 152.9, 149.8, 144.5, 143.4, 126.0, 123.3, 121.6, 121.3, 120.7, 120.4, 120.1, 109.0, 61.8, 52.5, 51.9, 14.4. HRMS (ESI) of C_17_H_16_BrN_3_O_4_S, *m/z*: calcd for [M+H]^+^ 319.9587 and 321.9566; found 319.9598 and 321.9576.

### 3.8. General Method for Hydrolysis of Carboxylic Esters **5**, **7** and **8**

KOH (215 mg, 3.8 mmol) was added to a solution of ester **5**, **7** or **8** (1 mmol) in 10 mL of EtOH and 10 mL of THF. The reaction mixture was stirred at 60 °C for 24 h. The formed precipitate was filtered off, washed with THF and dried under high vacuum.

### 3.9. Potassium 3-Amino-5-((6-bromo-1H-indol-1-yl)methyl)thiophene-2-carboxylate (MNS2)

The product was obtained according to the general method and isolated as light brown powder. Yield: 89%.

^1^H NMR (599 MHz, D_2_O) δ 7.51 (s, 1H, C*H*(Ar)), 7.40 (d, *J* = 8.4 Hz, 1H, 2C*H*(Ar)), 7.12 (m, 2H, 2C*H*(Ar)), 6.43 (d, *J* = 3.2 Hz, 1H, C*H*(Ar)), 6.25 (s, 1H, C*H*(Ar)), 5.04 (s, 2H, C*H*_2_). ^13^C NMR (151 MHz, D_2_O) δ 171.72, 149.38, 142.76, 136.47, 129.56, 127.34, 122.88, 122.39, 120.26, 114.96, 112.70, 110.90, 101.79, 44.85. HRMS (ESI) of C_14_H_10_BrKN_2_O_2_S, *m/z*: calcd for [M+H]^+^ 388.9356 and 390.9336; found 388.9361 and 390.9342.

### 3.10. Potassium 3-Amino-5-((6-bromo-1H-indazol-1-yl)methyl)thiophene-2-carboxylate (MNS3)

The product was obtained according to the general method and isolated as light brown powder. Yield: 90%.

^1^H NMR (300 MHz, D_2_O) δ 8.07–7.78 (m, 1H, C*H*(Ar)), 7.68−7.48 (m, 1H, C*H*(Ar)), 7.37 (dd, *J* = 8.5, 4.5 Hz, 1H, C*H*(Ar)), 7.14−6.81 (m, 1H, C*H*(Ar)), 6.52 (d, *J* = 3.3 Hz, 1H, C*H*(Ar)), 5.34 (d, *J* = 4.5 Hz, 2H, C*H*_2_). ^13^C NMR (75 MHz, D_2_O) δ 171.6, 149.4, 141.0, 139.7, 134.6, 124.7, 122.8, 122.5, 121.3, 121.20 112.2, 47.4. HRMS (ESI) of C_13_H_9_BrKN_3_O_2_S, *m/z*: calcd for [M+H]^+^ 389.9309 and 391.9289; found 389.9314 and 391.9295.

### 3.11. Potassium 3-Amino-5-((6-bromo-2H-indazol-2-yl)methyl)thiophene-2-carboxylate (MNS4)

The product was obtained according to the general method and isolated as light brown powder. Yield: 92%.

^1^H NMR (300 MHz, D_2_O) δ 8.01 (d, *J* = 1.0 Hz, 1H, C*H*(Ar)), 7.74−7.68 (m, 1H, C*H*(Ar)), 7.53 (dd, *J* = 8.6, 0.7 Hz, 1H, C*H*(Ar)), 7.19 (dd, *J* = 8.6, 1.6 Hz, 1H, C*H*(Ar)), 6.61 (s, 1H, C*H*(Ar)), 5.49 (s, 2H, (CH_2_)). ^13^C NMR (75 MHz, D_2_O) δ 160.7, 149.3, 141.0, 139.6, 134.5, 124.7, 122.7, 122.4, 121.3, 121.1, 112.1, 47.3. HRMS (ESI) of C_13_H_9_BrKN_3_O_2_S, *m/z*: calcd for [M+H]^+^ 389.9309 and 391.9289; found 389.9316 and 391.9295.

### 3.12. Methyl 5-Formyl-2-Methylfuran-3-Carboxylate

POCl_3_ (5.5 g, 35.7 mmol) was carefully added to ice-cooled DMF (5 mL). The solution was allowed to warm up to room temperature and was stirred for 1 h, followed by addition of methyl 2-methylfuran-3-carboxylate (5.0 g, 35.7 mmol). The reaction mixture was stirred for another 2 h at 100 °C. The mixture was cooled down to 0 °C, quenched with an aqueous solution of Na_2_CO_3_ (5 g/50 mL H_2_O) and extracted with CH_2_Cl_2_ (3 × 50 mL). The combined organic fractions were washed with brine and water, dried over MgSO_4_ and concentrated in vacuo. The residue was crystallized from Et_2_O. The product was obtained as yellow solid (4.3 g, 73% yield). Mp = 83–84 °C. The NMR data were in full agreement with previously published data [11].

^1^H NMR (300 MHz, CDCl_3_), δ: 9.57 (s, 1H, C*H*O), 7.48 (s, 1H, C*H*), 3.88 (s, 3H, OC*H*_3_), 2.70 (s, 3H, C*H*_3_). ^13^C NMR (75 MHz, CDCl_3_), δ: 177.1, 123.3, 122.3, 114.3, 112.3, 51.9, 14.3.

HRMS (ESI) of C_8_H_8_O_4_, *m/z*: calcd for [M+H]^+^ 169.0495; found 169.0501.

### 3.13. Methyl 5-(chloromethyl)-2-methylfuran-3-carboxylate (9)

Sodium borohydride (1.16 g, 30.64 mmol) was added to a solution of methyl 5-formyl-2-methylfuran-3-carboxylate(10) in methanol (70 mL) and CH_2_Cl_2_ (35 mL) at 0°C (4.30 g, 25.59 mmol). The mixture was stirred for 30 min at 0 °C and at rt for another 2 h. The resulting mixture was quenched with water and partitioned between water and CH_2_Cl_2_. The combined organic fractions were washed with brine and water, dried over MgSO_4_ and concentrated in vacuo. The crude product was purified on a silica gel column (eluent: petroleum ether−AcOEt, 2:1, Rf = 0.3). The product was obtained as colorless oil (3.93 g, 91% yield). The NMR data were in full agreement with previously published data [11].

^1^H NMR (300 MHz, CDCl_3_), δ: 6.52 (s, 1H, C*H*), 4.54 (s, 2H, C*H*_2_), 3.81 (s, 3H, OC*H*_3_), 2.56 (s, 3H, C*H*_3_), 2.02 (br.s, 1H, O*H*). ^13^C NMR (76 MHz, CDCl_3_), δ: 164.6, 159.4, 152.0, 113.8, 108.5, 57.0, 51.4, 13.8.

MsCl (5.30 g, 46.31 mmol) was added drop-wise to a mixture of methyl 5-(hydroxymethyl)-2-methylfuran-3-carboxylate (**11**) (3.93 g, 23.11 mmol) and Et_3_N (4.68 g, 46.31 mmol) dissolved in CH_2_Cl_2_ (40 mL). The solution was stirred for 4 h at rt, quenched with water and partitioned between water and CH_2_Cl_2_. The combined organic fractions were washed with brine and water, dried over MgSO_4_ and concentrated in vacuo. The crude product was purified on a silica gel column (eluent: petroleum ether−AcOEt, 10:1, (Rf = 0.35). The product was obtained as colorless oil (3.27 g, 75% yield). The NMR data were in full agreement with previously published data [11].

^1^H NMR (300 MHz, CDCl_3_), δ: 6.61 (s, 1H, C*H*), 4.52 (s, 2H, C*H*_2_), 3.82 (s, 3H, OC*H*_3_), 2.59 (s, 3H, C*H*_3_). ^13^C NMR (75 MHz, CDCl_3_), δ: 164.0, 160.2, 148.0, 114.3, 110.5, 51.4, 37.1, 13.8. HRMS (ESI) of C_8_H_9_ClO_3_, *m/z*: calcd for [M+H]^+^ 189.0313; found 189.0318.

### 3.14. Methyl 5-((6-Bromo-1H-indazol-1-yl)methyl)-2-methylfuran-3-carboxylate (10) and Methyl 5-((6-Bromo-2H-indazol-2-yl)methyl)-2-methylfuran-3-carboxylate (11)

6-Bromoindole (150 mg, 0.76 mmol) was added to a stirred suspension of NaH (60% dispersion in oil, 27.4 mg, 45.7 mmol) in dry DMF (3 mL). The mixture was stirred for 4 h, and methyl 5-(chloromethyl)-2-methylfuran-3-carboxylate (**9**) (215 mg, 1.14 mmol) was added. The solution was stirred at rt overnight. The mixture was quenched with water and partitioned between water and EtOAc. The combined organic fractions were washed with brine and water, dried over MgSO_4_ and concentrated in vacuo. The crude product was purified on a silica gel column (eluent: petroleum ether−AcOEt, 5:1 → 3:1, Rf = 0.25 and 0.15 for 10 and 11 in petroleum ether−AcOEt = 5:1) to afford the target compounds as pale-yellow waxy solids (150 and 44 mg, 56% and 17% yield).

Major (**10**)

^1^H NMR (300 MHz, CDCl_3_) δ 7.97 (d, *J* = 0.8 Hz, 1H, C*H*(Ar)), 7.66 (s, 1H, C*H*(Ar)), 7.59 (t, *J* = 6.9 Hz, 1H, C*H*(Ar)), 7.32–7.19 (m, 1H, C*H*(Ar)), 6.55 (s, 1H, C*H*(Ar)), 5.43 (s, 2H, CH_2_), 3.78 (s, 3H, OCH_3_), 2.51 (s, 3H, CH_3_). ^13^C NMR (75 MHz, CDCl_3_) δ 164.1, 159.7, 147.2, 140.2, 133.9, 124.6, 123.2, 122.3, 121.1, 114.2, 112.1, 109.6, 51.4, 45.8, 13.8. HRMS (ESI) of C_15_H_13_BrN_2_O_3_, *m/z*: calcd for [M+H]^+^ 349.0182 and 351.0163; found 349.0183 and 351.0164.

Minor (**11**)

^1^H NMR (300 MHz, CDCl_3_) δ 7.92 (s, 1H, C*H*(Ar)), 7.88 (s, 1H, C*H*(Ar)), 7.50 (d, *J* = 8.9 Hz, 1H, C*H*(Ar)), 7.14 (dd, *J* = 8.9, 1.6 Hz, 1H, C*H*(Ar)), 6.72 (s, 1H, C*H*(Ar)), 5.49 (s, 1H, C*H*_2_), 3.81 (s, 3H, OC*H*_3_), 2.54 (s, 3H, C*H*_3_). ^13^C NMR (75 MHz, CDCl_3_) δ 163.9, 160.3, 149.6, 146.0, 125.7, 123.2, 121.6, 120.6, 120.2, 120.0, 114.4, 111.1, 51.4, 49.9, 13.8. HRMS (ESI) of C_15_H_13_BrN_2_O_3_, *m/z*: calcd for [M+H]^+^ 349.0182 and 351.0163; found 349.0185 and 351.0167.

### 3.15. 5-((6-Bromo-1H-indazol-1-yl)methyl)-2-methylfuran-3-carboxylic Acid (MNS5)

An aqueous solution of NaOH (86 mg, 2.15 mmol in 4 mL of water) was added to methyl 5-((6-bromo-1*H*-indol-1-yl)methyl)-2-methylfuran-3-carboxylate (**10**) (150 mg, 0.43 mmol) dissolved in 8 mL of methanol. The mixture was refluxed for 3 h, cooled down to room temperature, and then, MeOH was partially removed in vacuo. The remaining aqueous solution was treated with 10% aq. HCl (to reach pH = 1). The resulting precipitate was filtered out, washed with water and dried in high vacuum to afford the product as a colorless solid (103 mg, 72% yield).

^1^H NMR (300 MHz, DMSO-d6) δ 12.56 (s, 1H, O*H*), 8.13 (s, 1H, C*H*(Ar)), 8.12 (s, 1H, C*H*(Ar)), 7.74 (d, *J* = 8.5 Hz, 1H, C*H*(Ar)), 7.29 (dd, *J* = 8.5, 1.5 Hz, 1H, C*H*(Ar)), 6.63 (s, 1H, C*H*(Ar)), 5.62 (s, 2H, C*H*_2_), 2.42 (s, 3H, CH_3_). ^13^C NMR (75 MHz, DMSO-d6) δ 165.1, 159.0, 148.4, 140.3, 134.3, 124.5, 123.4, 123.2, 120.54, 112.9, 110.2, 45.2, 13.8. HRMS (ESI) of C_14_H_11_BrN_2_O_3_, *m/z*: calcd for [M+H]^+^ 335.9978; found 335.9983.

### 3.16. 5-((6-Bromo-2H-indazol-2-yl)methyl)-2-methylfuran-3-Carboxylic Acid (MNS6)

An aqueous solution of NaOH (44 mg, 0.13 mmol in 1 mL of water) was added to the solution of methyl 5-((6-bromo-1*H*-indol-1-yl)methyl)-2-methylfuran-3-carboxylate (**11**) (25 mg, 0.63 mmol) dissolved in 2 mL of methanol. The mixture was refluxed for 3 h and cooled down to room temperature; then, MeOH was removed in vacuo. The remaining aqueous solution was treated with 10% aq. HCl (to reach pH = 1). The precipitate was filtered out, washed with water and dried in high vacuum to afford the product as a colorless solid (30 mg, 71% yield).

^1^H NMR (300 MHz, DMSO-d6) δ 12.56 (s, 1H, O*H*), 8.47 (s, 1H, C*H*(Ar)), 7.86 (s, 1H, C*H*(Ar)), 7.70 (d, *J* = 8.9 Hz, 1H, C*H*(Ar)), 7.14 (dd, *J* = 8.9, 1.5 Hz, 1H, C*H*(Ar)), 6.73 (s, 1H, C*H*(Ar)), 5.65 (s, 2H, CH_2_), 2.46 (s, 3H, CH_3_). ^13^C NMR (75 MHz, DMSO-d6) δ 165.0, 159.5, 149.2, 147.6, 125.6, 125.1, 123.5, 120.6, 119.8, 119.4, 115.0, 111.2, 49.3, 13.8. HRMS (ESI) of C_14_H_11_BrN_2_O_3_, *m/z*: calcd for [M+H]^+^ 335.9978; found 335.9981.

### 3.17. Estimation of the Minimum Inhibitory Concentration (MIC) of Gentamycin in B. subtilis Cells

Standardized MIC was determined using the microdilution method, as recommended by the Clinical and Laboratory Standards Institute (CLSI) [28]. Twofold serial dilutions of the antibiotic were prepared in 100 μL of lysogenic broth (LB) (Sigma-Aldrich, St. Louis, MO, USA) supplemented with 50 μM CGL. The *B. subtilis* inoculate (100 μL) contained 1.0 × 10^6^ CFU/mL. The minimum inhibitory concentration (MIC) was defined as the lowest antibiotic concentration that prevented turbidity in the inoculate following incubation at 37 °C for a duration of 24 h. Gentamycin was used at 0.1 μg/mL (10% of MIC) in further experiments.

Gentamicin-potentiating activity testing of the compounds in *B. subtilis*. The growth curves of *B. subtilis* were obtained with a Bioscreen C automated growth curve analysis system (Oy Growth Curves Ab, Finland, Helsinki). The B. subtilis strain 168 was grown overnight in LB at 37 °C and diluted with fresh medium (1:100) containing the antibiotic and a test compound as specified in the text and figure captions. Aliquots (150 μL) of each mixture were added into plate wells in triplicate and incubated with continuous agitation at 37 °C. The optical density at 600 nm (OD_600_) was recorded automatically for 24 h. The results were averaged over three replicate experiments to plot the time dependences of bacterial growth.

The generation of hydrogen sulfide in *B. subtilis* cells was monitored according to the published protocol using lead acetate as a colorimetric sensor [29]. The inner surface of a cell culture flask was equipped above the level of the liquid with a standardized paper strip soaked with 2% aq. Pb(OAc)_2_. Overnight cultures were diluted with LB (1:50) and incubated at 37 °C under aeration conditions for 16–18 h. The test compounds (**MNS1** and **NL2**) were used at 32 μM; LB without supplements was used as a negative control. Paper strips stained with the formed PbS precipitate were scanned using an AlphaImager gel imaging system (BioTechne, USA, Minneapolis, Minnesota). The results were normalized to the optical density of the culture.

### 3.18. Cell Culture

HEK293T cells were grown in the DMEM:F12 (2:1) medium supplemented with 10% FBS (BioSera, Cholet, France), 2 mM L-glutamine, 100 U/mL penicillin and 100 μg/mL streptomycin in the presence of 5% CO_2_ at 37 °C. Cells were re-seeded every three days at a ratio of 1:3 or 1:5.

### 3.19. MTT Test

Stock solutions were diluted in DMSO (BioReagent of molecular biology grade, Sigma, St. Louis, MO, USA), and the final DMSO concentration was 1%. After 24 h (30–40% monolayer), the solutions of the studied compounds diluted at different concentrations were introduced to the culture medium. After the incubation at 37 °C in 5% CO_2_ for 72 h, a freshly prepared solution of thiazolyl blue tetrazolium bromide at a final concentration of 0.4 mg/mL was added to each well of the plate. Incubation was performed at +37 °C for 2 h. The medium containing unreacted MTT was removed via aspiration. The formed formazan dye was dissolved in 100 µL of isopropanol added to each well of the plate. The result of the analysis was evaluated using a Tecan Spark spectrophotometer (absorbance at 544 nm).

## 4. Conclusions

In this study, we expanded the range of indole-based potentiators, starting with the synthesis of **MNS2**, a thiophene analog of **NL2**. The developed method for **MNS2** was applied to obtain a range of new indazole-based gentamicin potentiators containing both the thiophene residue (potassium salts of 3-amino-5-((6-bromo-1*H*-indol-1-yl)methyl)thiophene-2-carboxylate (**MNS2**) and 3-amino-5-((6-bromo-1H-indazol-1-yl)methyl)thiophene-2-carboxylate (**MNS3**), 3-amino-5-((6-bromo-2H-indazol-2-yl)methyl)thiophene-2-carboxylate (**MNS4**)) and their analogs with the furan moiety (3-amino-5-((6-bromo-1H-indazol-1-yl)methyl)furan-2-carboxylic acid (**MNS5**) and 3-amino-5-((6-bromo-2H-indazol-2-yl)methyl)furan-2-carboxylic acid (**MNS6**)). All compounds were tested in *B. subtilis* cells as structurally potential inhibitors of CGL. The antibacterial effect of gentamicin in cells was enhanced, and bacterial H_2_S production activity was also altered. Balanced activity was found for the **MNS3** and **MNS4** compounds, showing both potentiation for gentamicin and kanamycin and moderate cytotoxicity in HEK273T. However, their influence on H_2_S production is yet to be explained. The developed methods for synthesis can serve as a basis for the design of novel generations of antibiotic potentiators.

## Figures and Tables

**Table 1:** MIC (potentiators + antibiotics) and CC_50_ of the synthesized **MNS2**–**MNS5** and their parent compound **MNS1**.

	MNS1	MNS2	MNS3	MNS4	MNS5	MNS6
MIC with Gm (*B. subtilis*) *^a^*	~50 μM	~100 μM	~90 μM	~60 μM	~60 μM	~100 μM
MIC with Km (*B. subtilis*) *^b^*	100	–	90	70	100	–
CC_50_ of compounds (HEK273T) *^c^*	30 μM	25 μM	190 μM	87 μM	250 μM	260 μM

*^a^* MIC values in the presence of 0.1 µg Gm (MIC_Gm_ = 1 µg/mL) were calculated by varying the concentration of a potentiator with an interval of 10 μM. *^b^* MIC values in the presence of 0.25 µg Km (MIC_Km_ = 2 µg/mL) were calculated by varying the concentration of a potentiator with an interval of 10 μM. *^c^* CC_50_ value is the concentration of a compound required to inhibit 50% cell culture growth.

## Data Availability

Data are contained within the article and Supplementary Materials.

## Supplementary Materials

The following supporting information can be downloaded at: https://www.mdpi.com/article/10.3390/molecules30020388/s1, NMR spectral data for compounds.
