# Supplementary material for: 6-Bromoindole- and 6-Bromoindazole-Based Inhibitors of Bacterial Cystathionine γ-Lyase Containing 3-Aminothiophene-2-Carboxylate Moiety
Source: Molecules. 2025 Jan 17;30(2):388. doi: 10.3390/molecules30020388 (PMC11767297; doi:10.3390/molecules30020388)

ELECTRONIC SUPPLEMENTARY MATERIAL

6-Bromoindole- and 6-bromoindazole-based inhibitors of bacterial cystathionine γ-lyase containing 3-aminothiophene-2-carboxylate moiety

Roman A. Novikov [1,2,*], Dmitry N. Platonov [2], Alexander Yu. Belyy [2], Konstantin V. Potapov [1,2], Maxim A. Novikov [1,2], Yury V. Tomilov [2], Olga I. Kechko [1], Tatiana A. Seregina [1], Pavel N. Solyev [1,*], Vladimir A. Mitkevich [1]

[1] Engelhardt Institute of Molecular Biology of the Russian Academy of Sciences, 32 Vavilov St., Moscow 119991, Russia; kospotapov@yandex.ru (K.V.P.); novikovfff@bk.ru (R.A.N.); manovikov@ioc.ac.ru (M.A.N.) solyev@gmail.com (P.N.S.); olga.kechko@gmail.com (O.I.K.); tatyana.s82@gmail.com (T.A.S.); mitkevich@gmail.com (V.A.M.);

[2] Zelinsky Institute of Organic Chemistry of the Russian Academy of Sciences, 47 Leninsky avenue, 119991 Moscow, Russia; dmplatonov@rambler.ru (D.N.P); duospirit@gmail.com (A.Y.B.); kospotapov@yandex.ru (K.V.P.); manovikov@ioc.ac.ru (M.A.N.); novikovfff@bk.ru (R.A.N.); tom@ioc.ac.ru (Y.V.T.);

* Correspondence: solyev@gmail.com (P.N.S.); Tel.: +7-499-135-60-65; novikovfff@bk.ru (R.A.N.)

NMR spectral data for compounds

^{1}H NMR (300 MHz) in $CDCl_3$

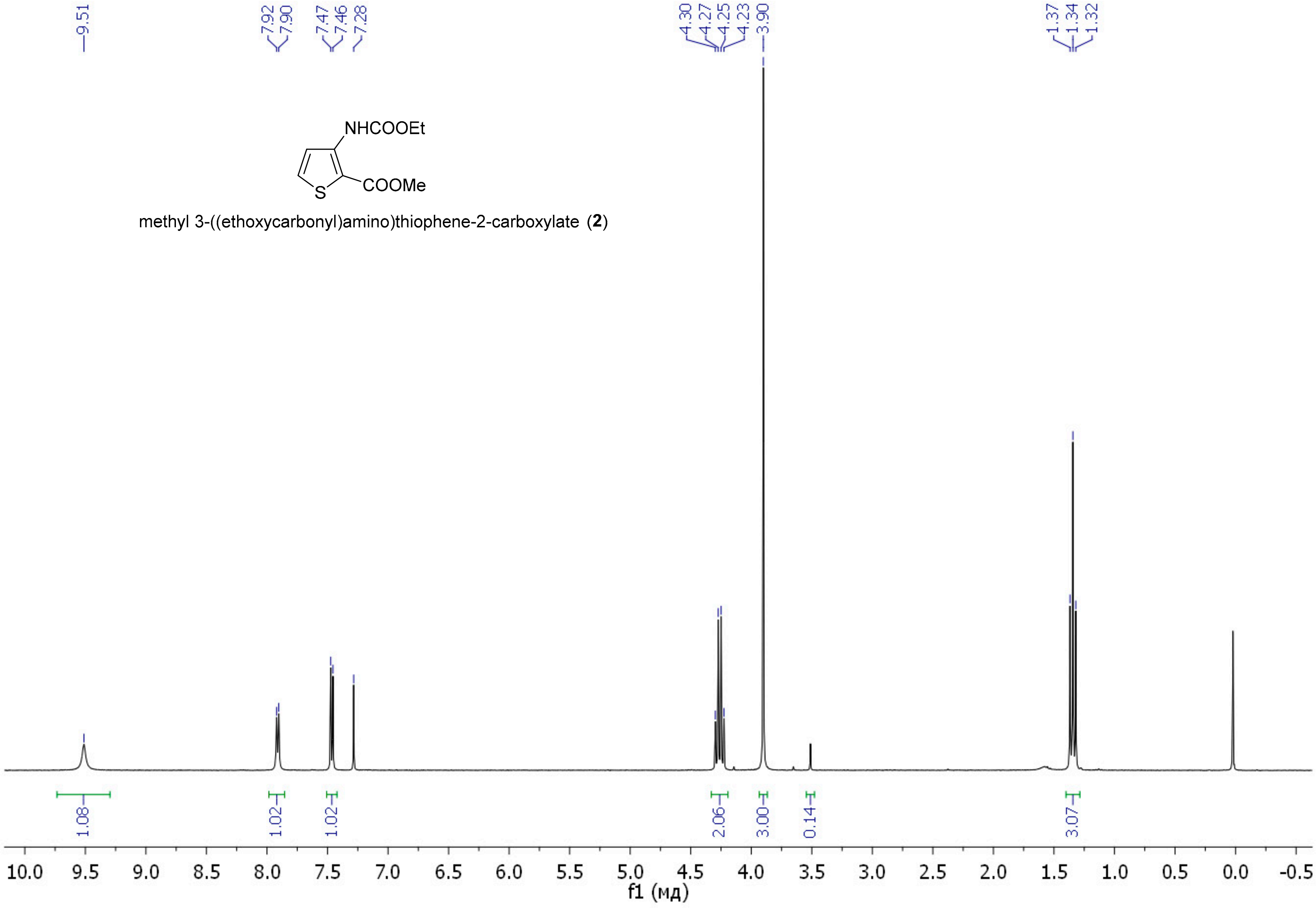

^{1}H NMR (300 MHz) in $CDCl_3$

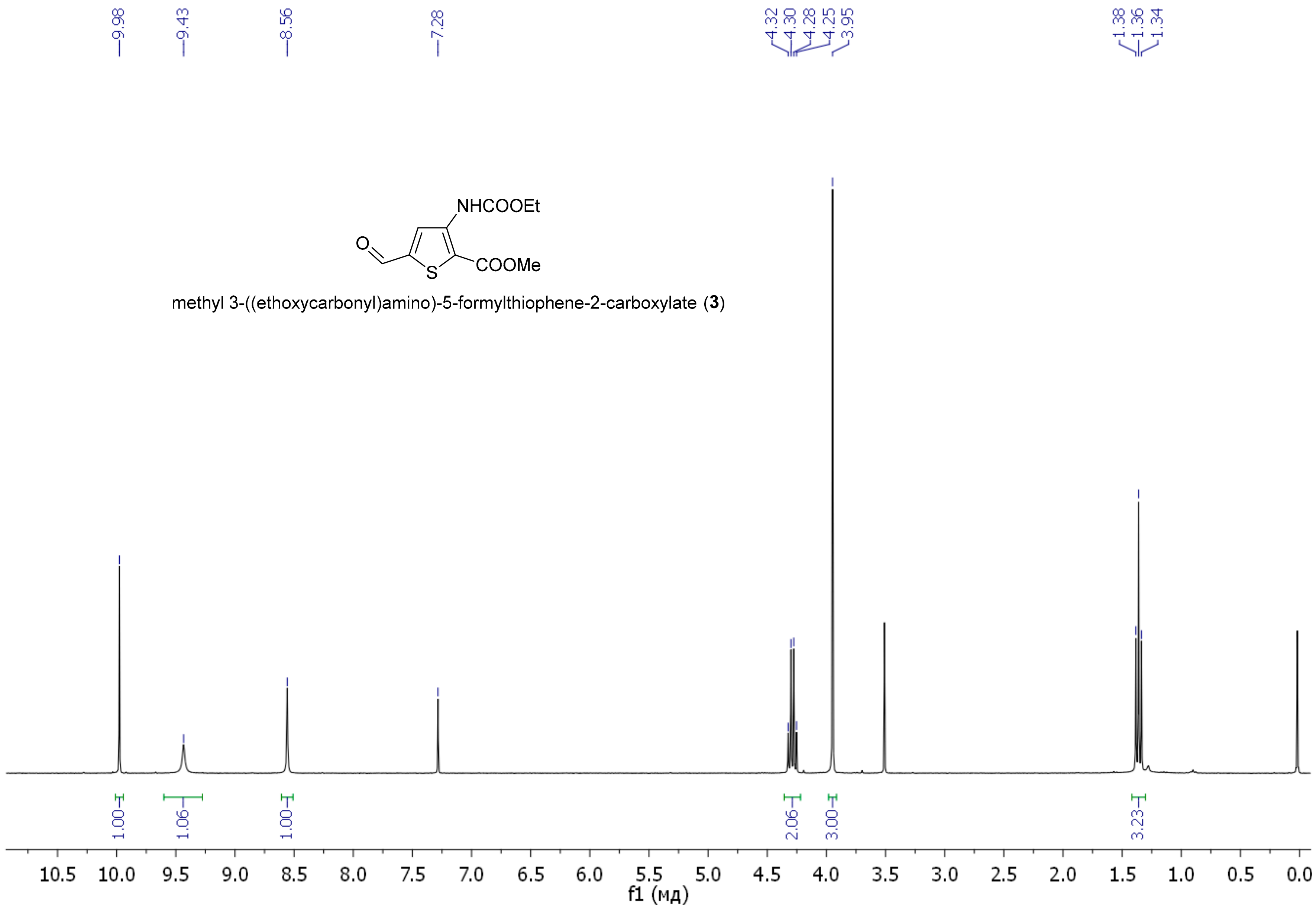

methyl 3-((ethoxycarbonyl)amino)-5-formylthiophene-2-carboxylate (**3**)

^{13}C NMR (75.5 MHz) in $CDCl_3$

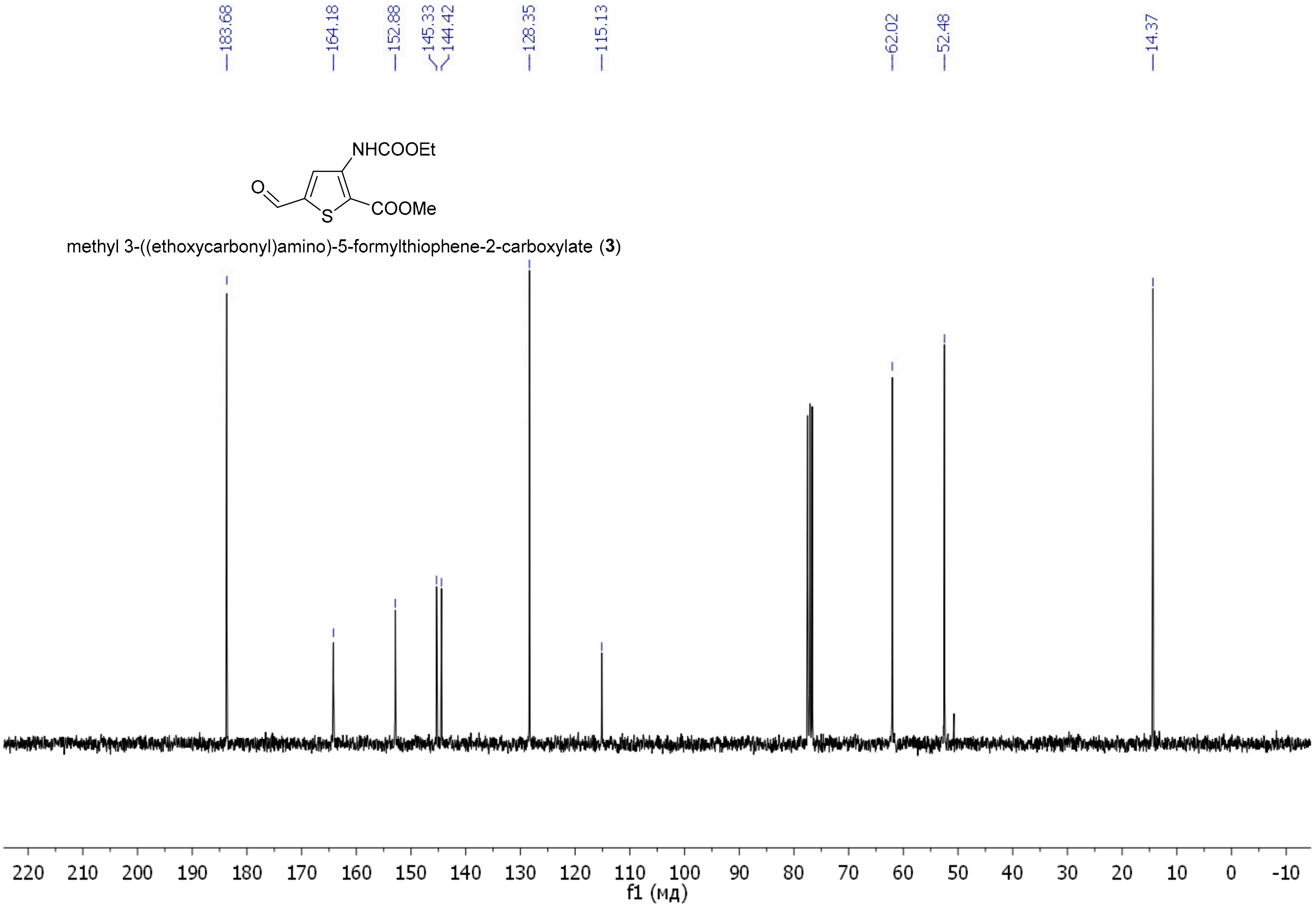

^{1}H NMR (300 MHz) in $CDCl_3$

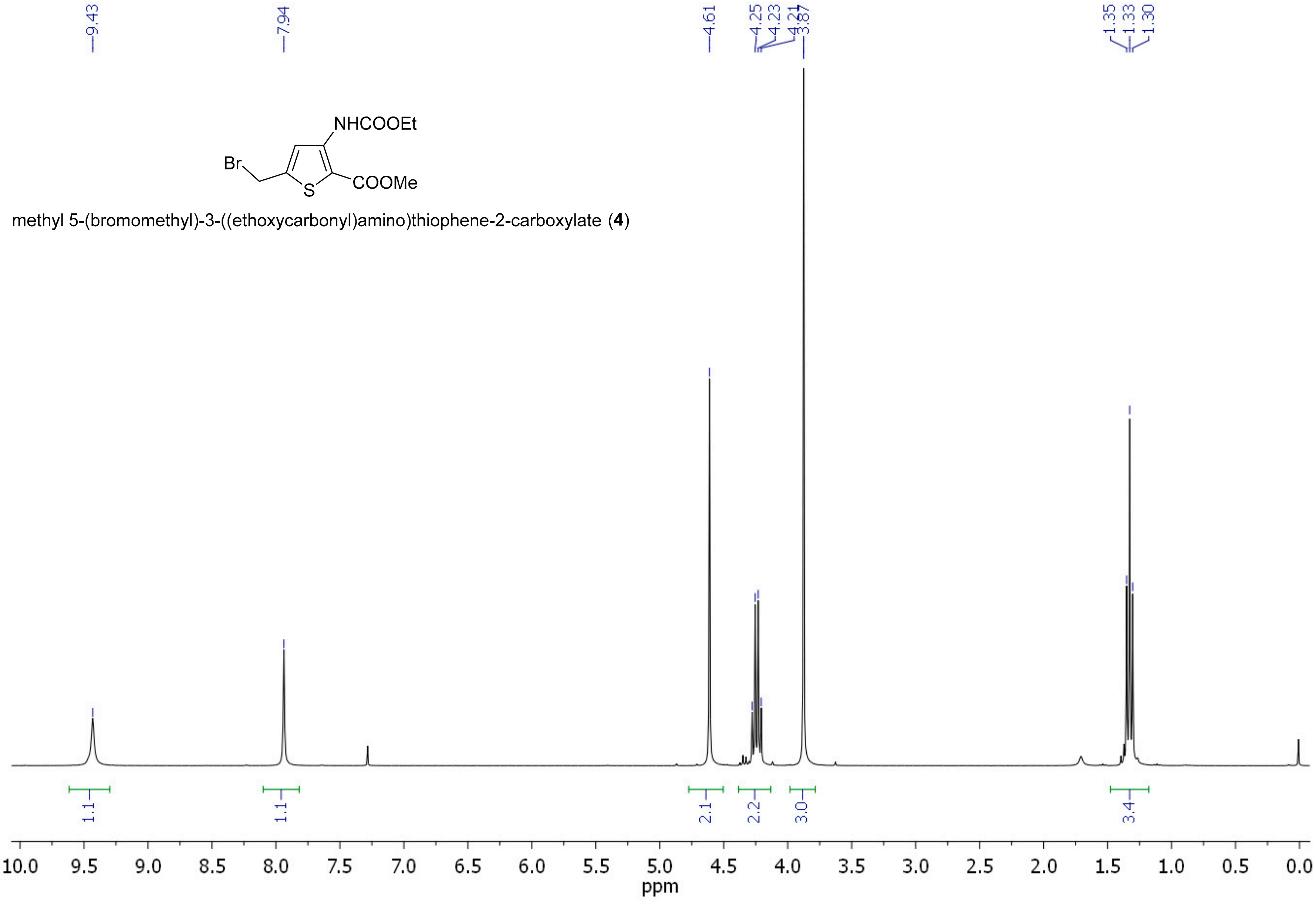

methyl 5-(bromomethyl)-3-((ethoxycarbonyl)amino)thiophene-2-carboxylate (**4**)

^{13}C NMR (75.5 MHz) in $CDCl_3$

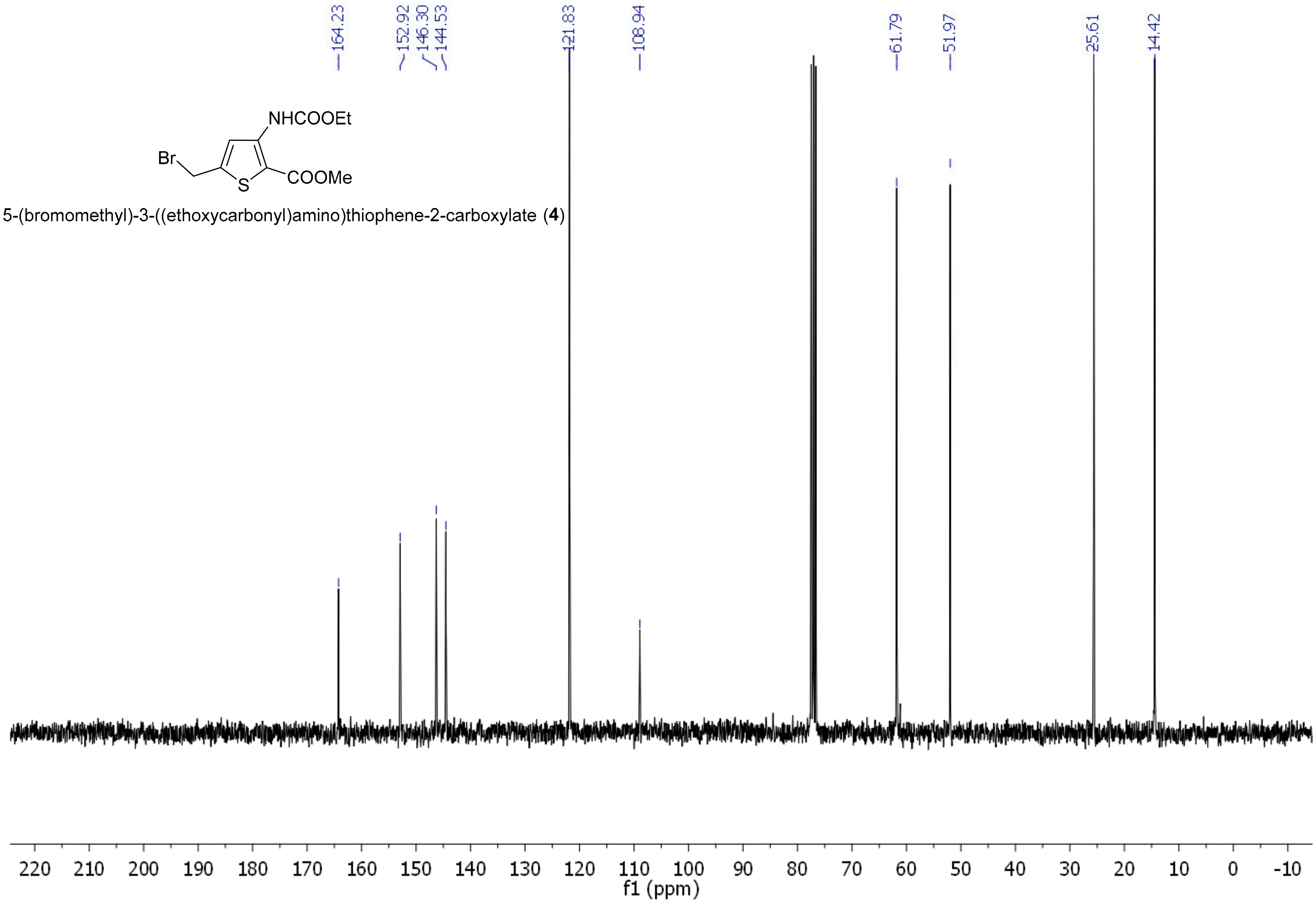

methyl 5-(bromomethyl)-3-((ethoxycarbonyl)amino)thiophene-2-carboxylate (**4**)

^{1}H NMR (300 MHz) in $CDCl_3$

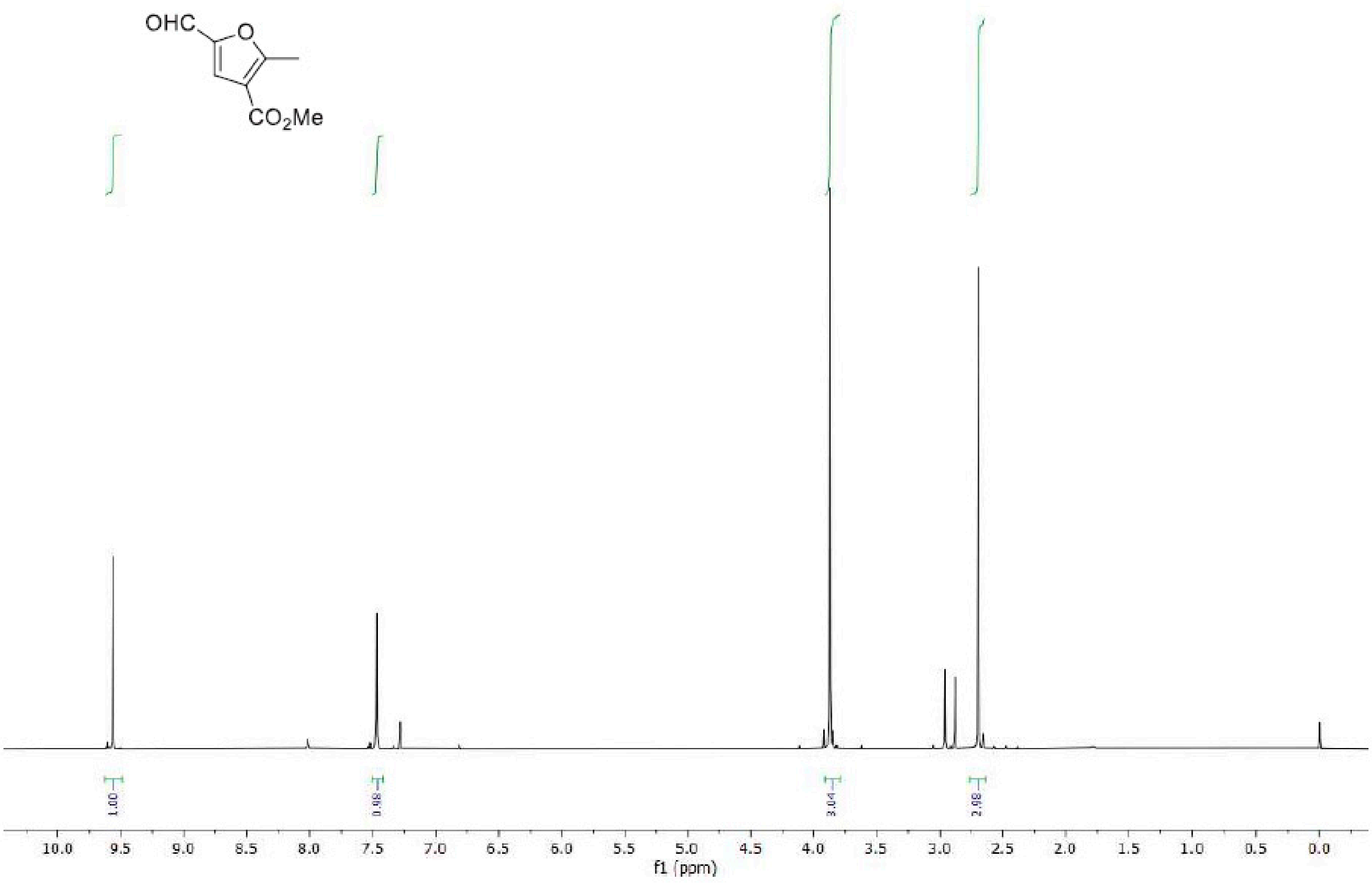

^{1}H NMR (300 MHz) in $CDCl_3$

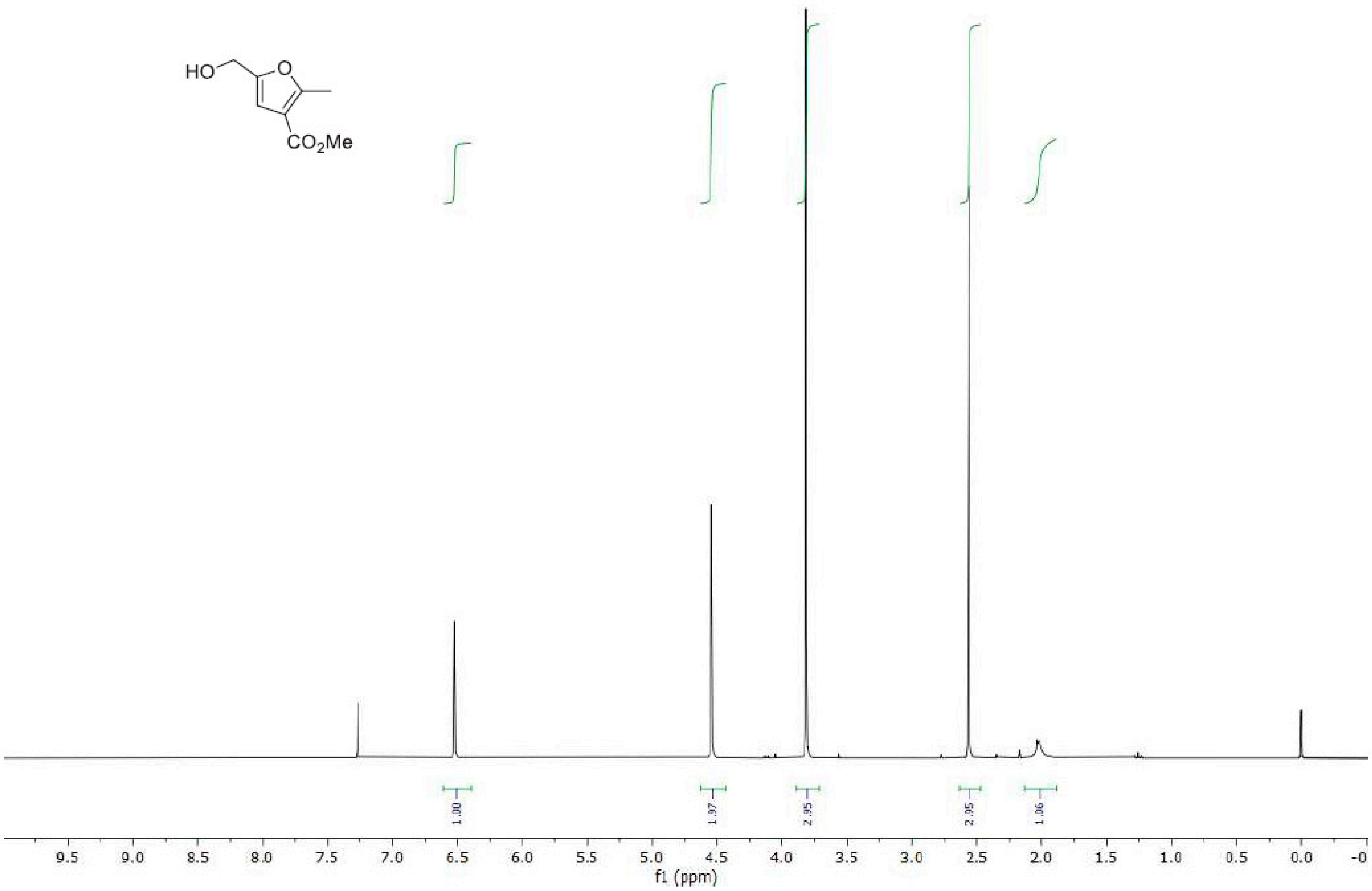

^{13}C NMR (75.5 MHz) in $CDCl_3$

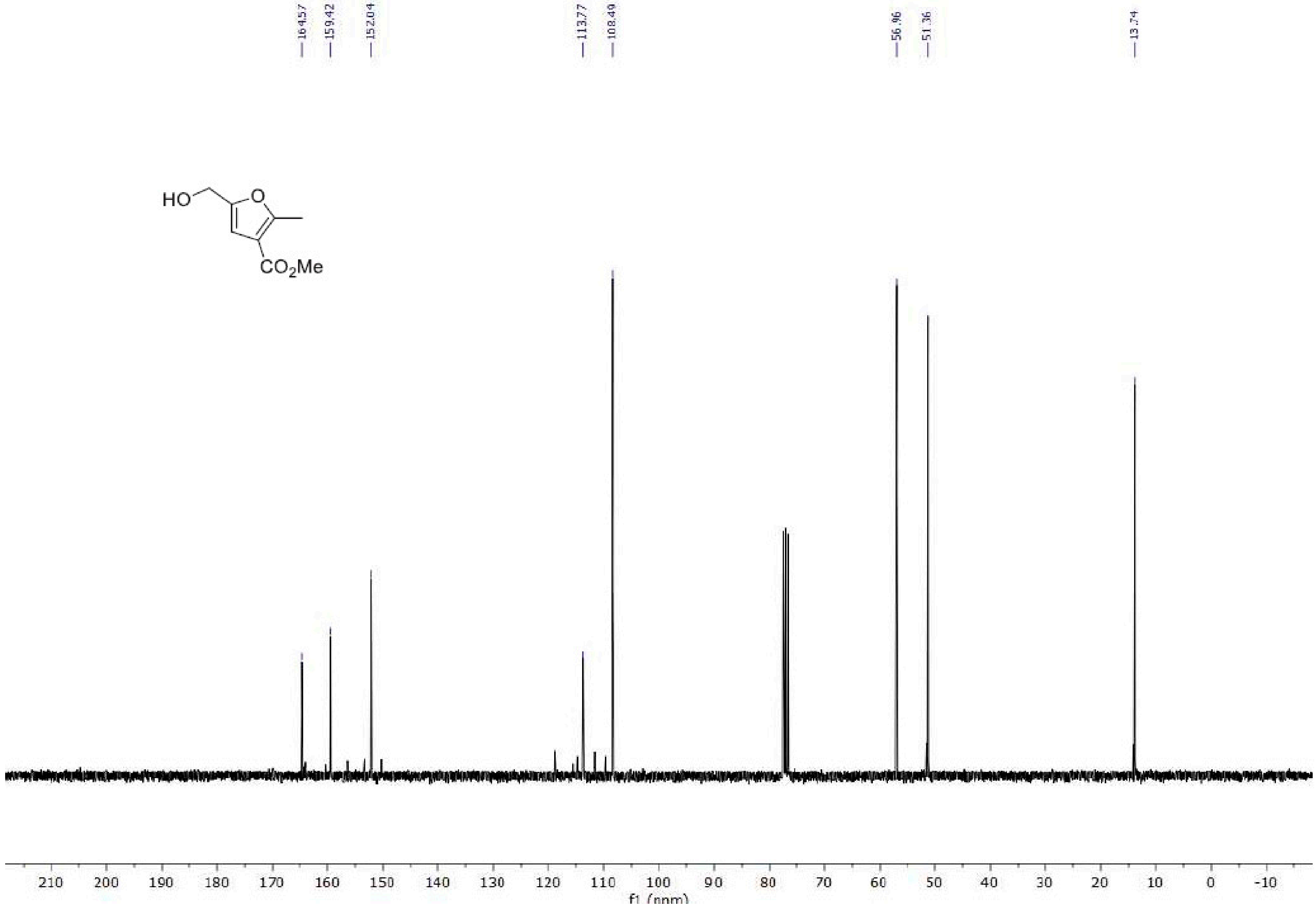

^{1}H NMR (300 MHz) in $CDCl_3$

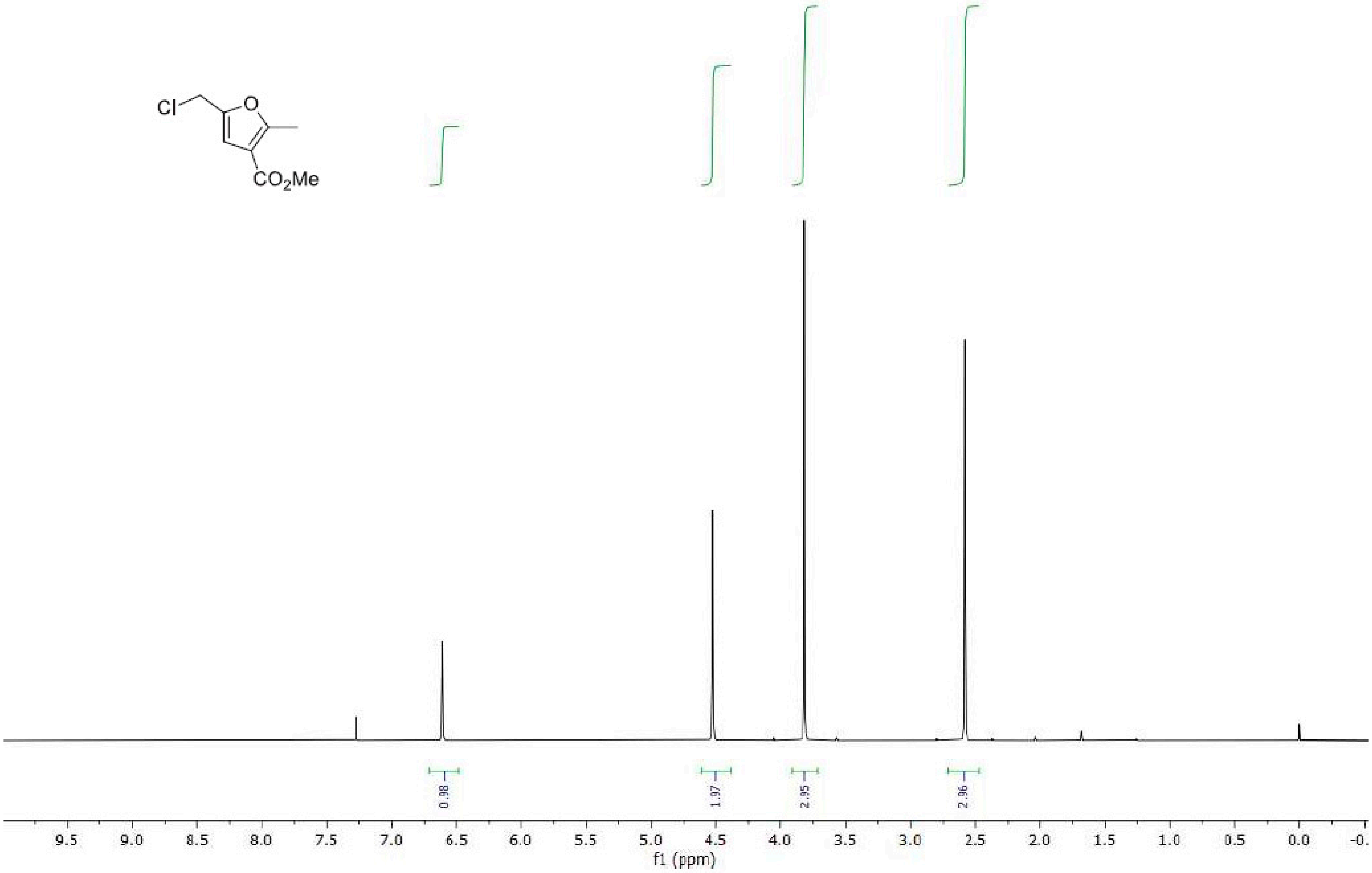

^{13}C NMR (75.5 MHz) in $CDCl_3$

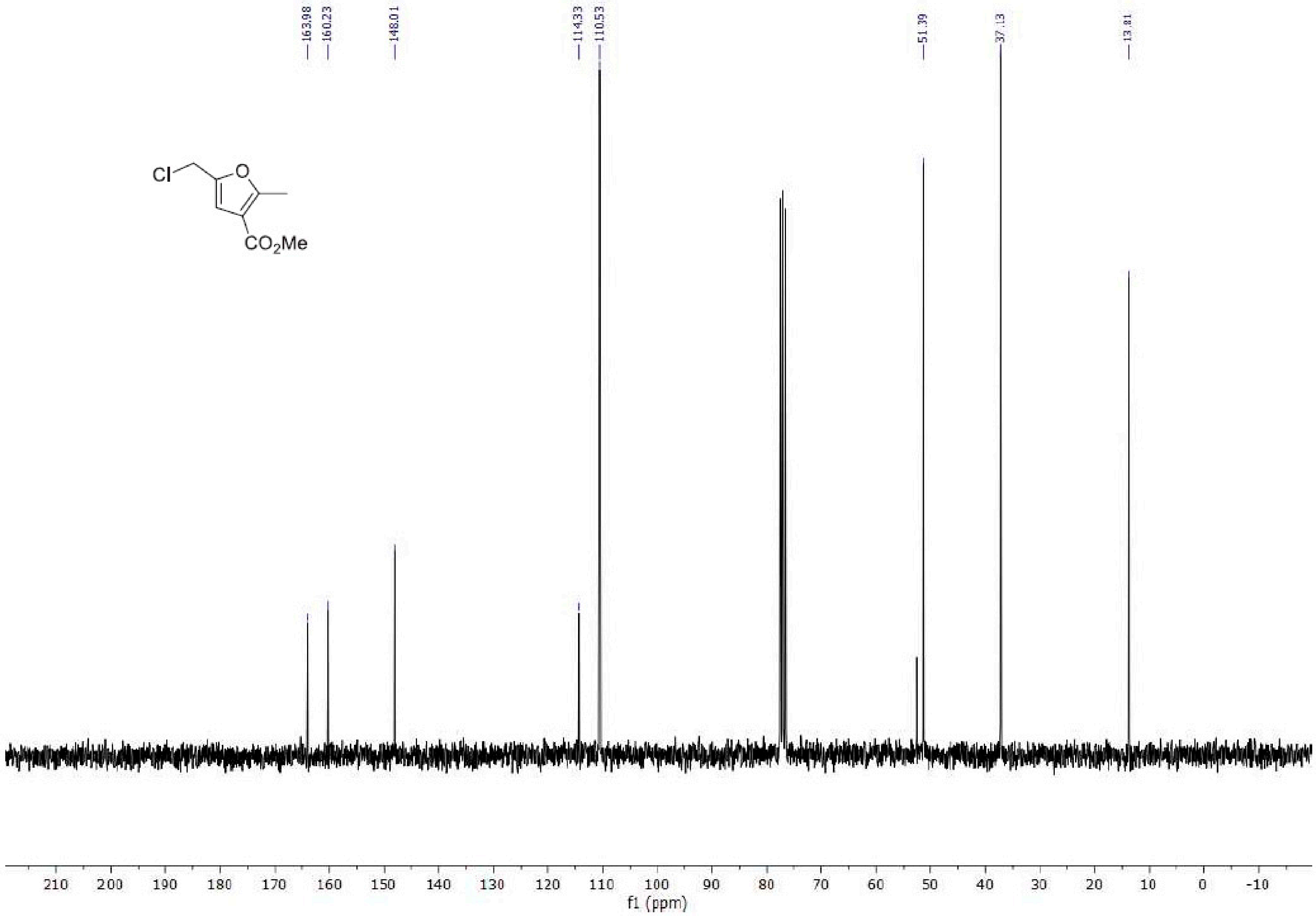

^{1}H NMR (300 MHz) in DMSO-d6

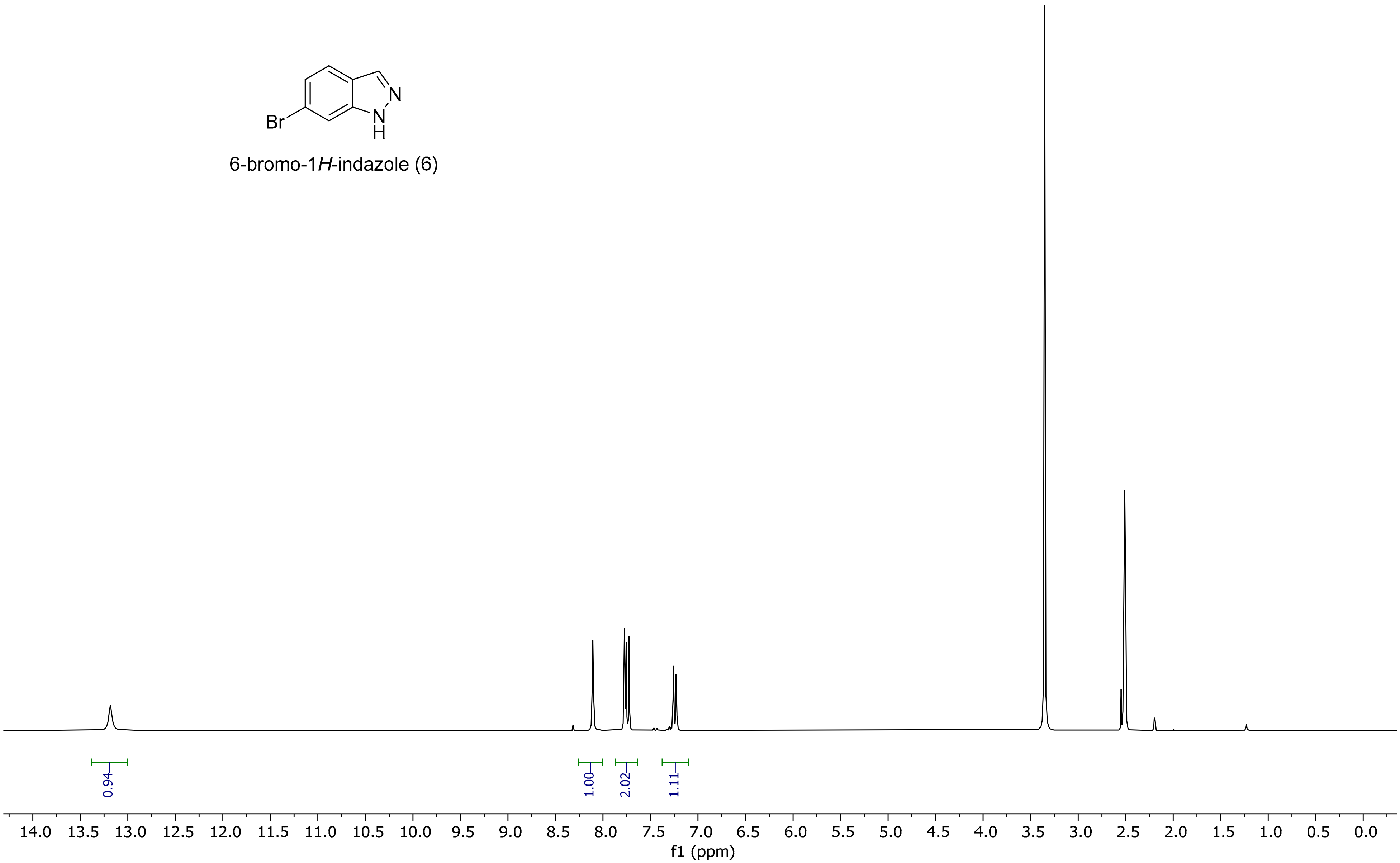

6-bromo-1*H*-indazole (6)

^{1}H NMR (300 MHz) in $CDCl_3$

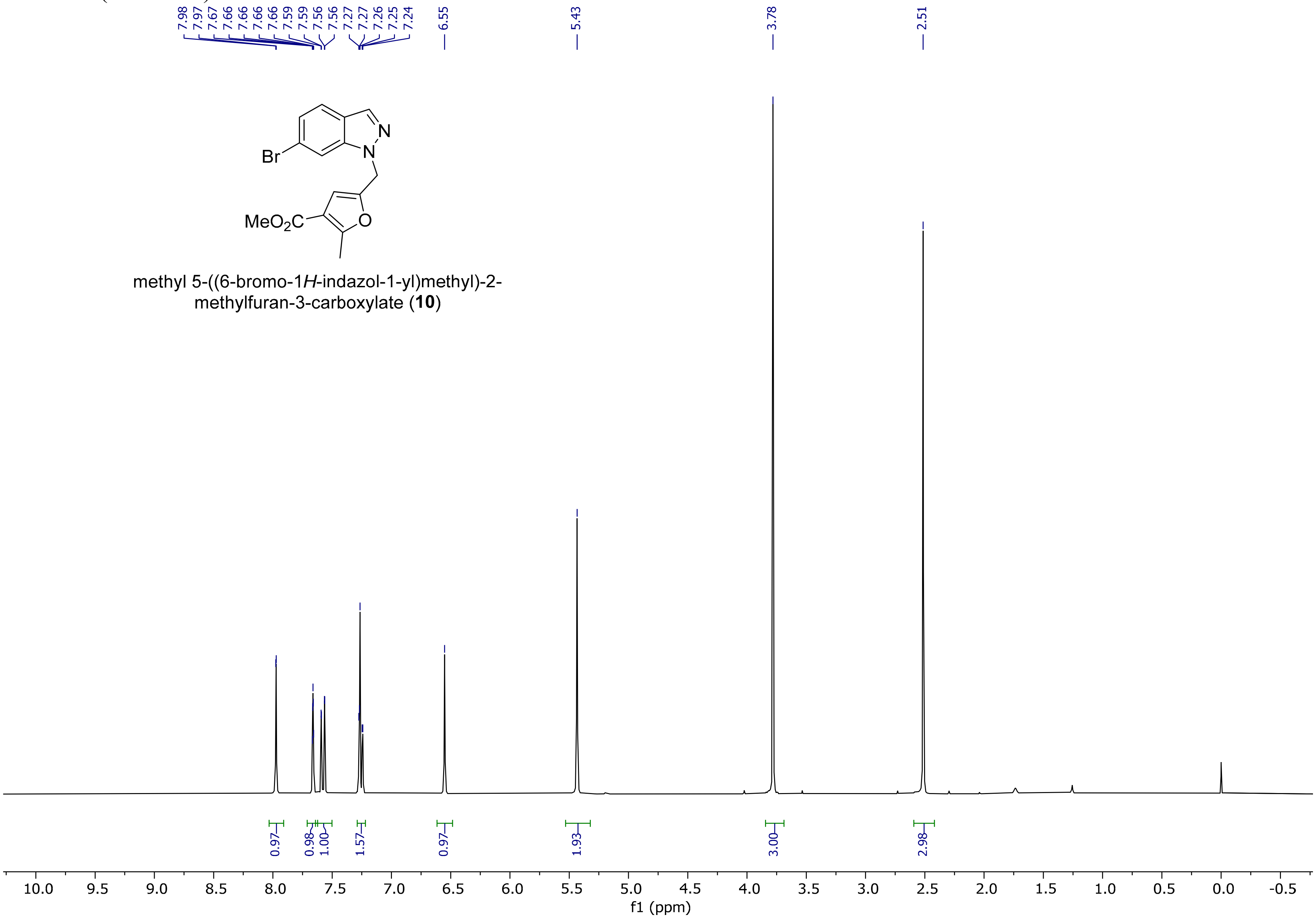

methyl 5-((6-bromo-1*H*-indazol-1-yl)methyl)-2-methylfuran-3-carboxylate (**10**)

^{13}C NMR (75.5 MHz) in $CDCl_3$

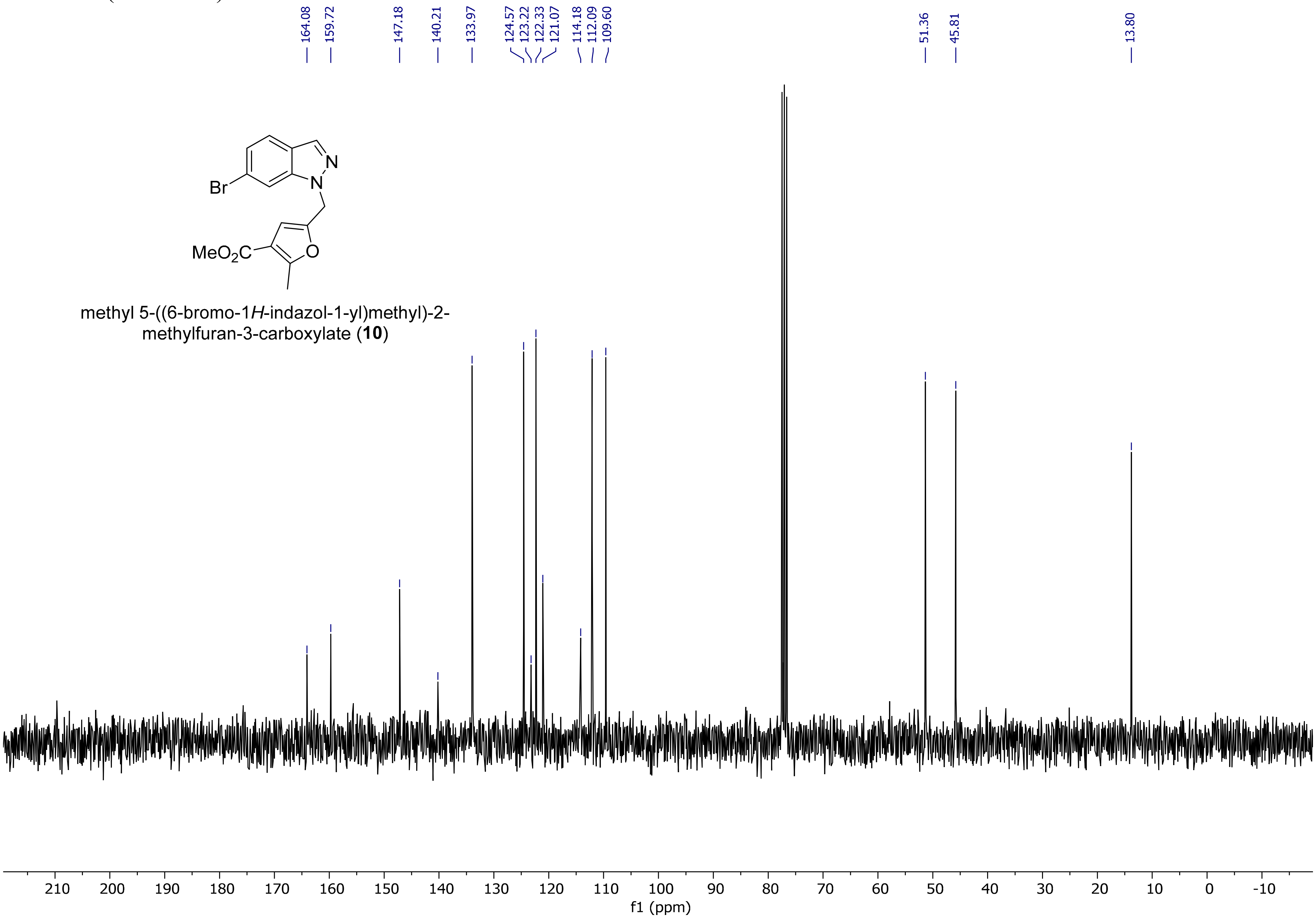

methyl 5-((6-bromo-1*H*-indazol-1-yl)methyl)-2-methylfuran-3-carboxylate (**10**)

^{1}H NMR (300 MHz) in $CDCl_3$

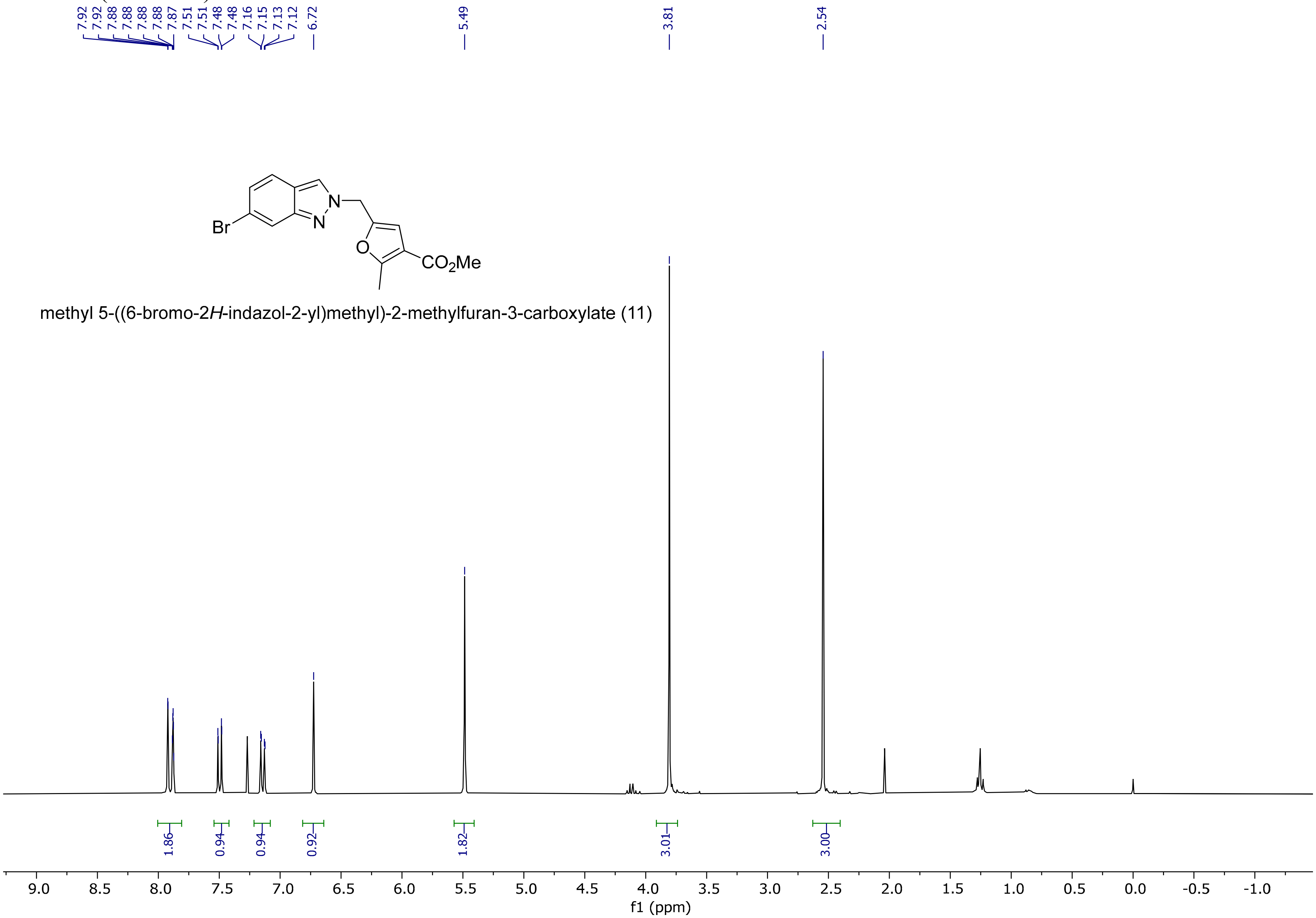

methyl 5-((6-bromo-2*H*-indazol-2-yl)methyl)-2-methylfuran-3-carboxylate (11)

^{13}C NMR (75.5 MHz) in $CDCl_3$

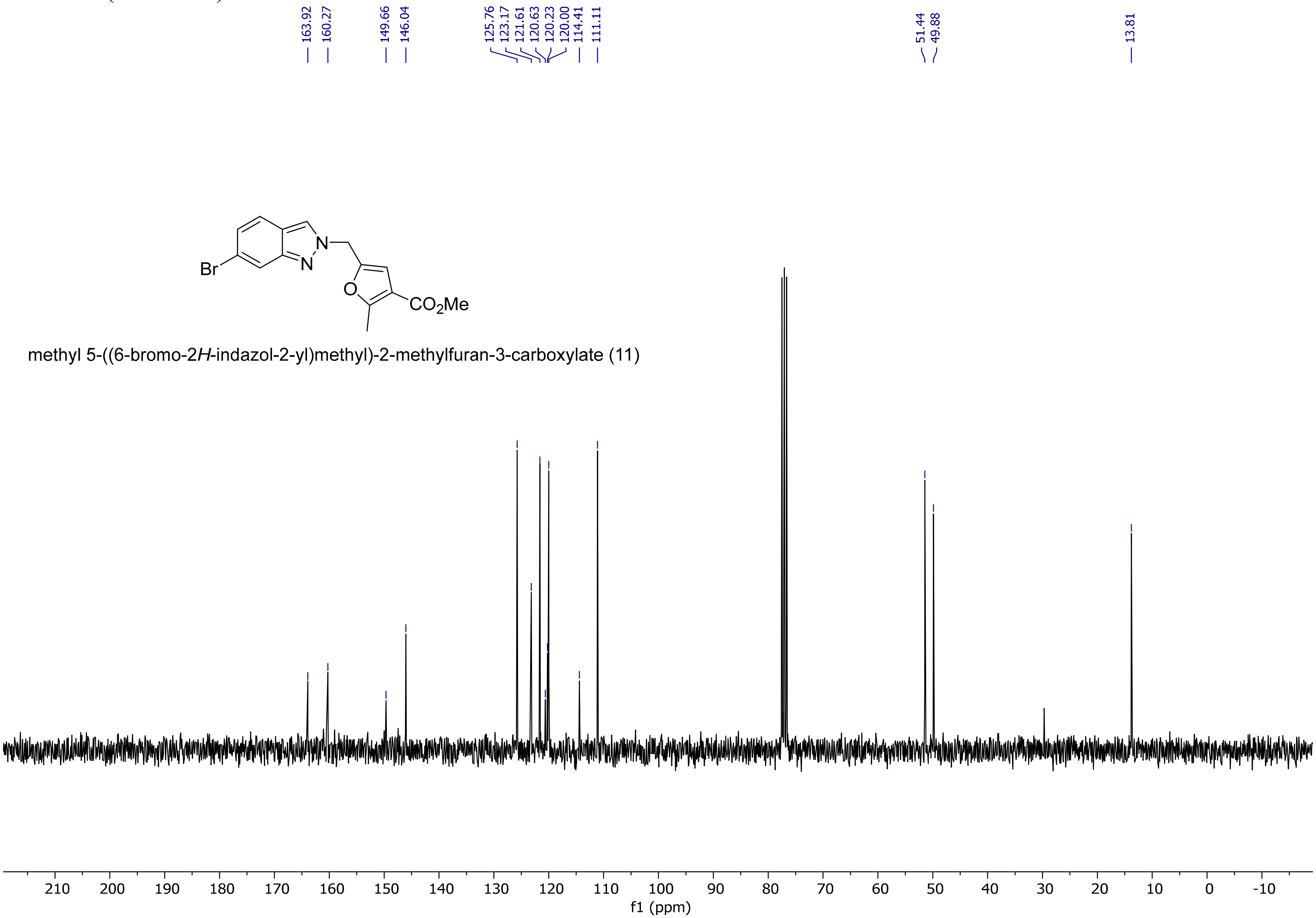

methyl 5-((6-bromo-2*H*-indazol-2-yl)methyl)-2-methylfuran-3-carboxylate (11)

^{1}H NMR (300 MHz) in DMSO-d6

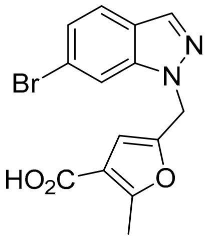

5-((6-bromo-1*H*-indazol-1-yl)methyl)-2-methylfuran-3-carboxylic acid (**MNS5**)

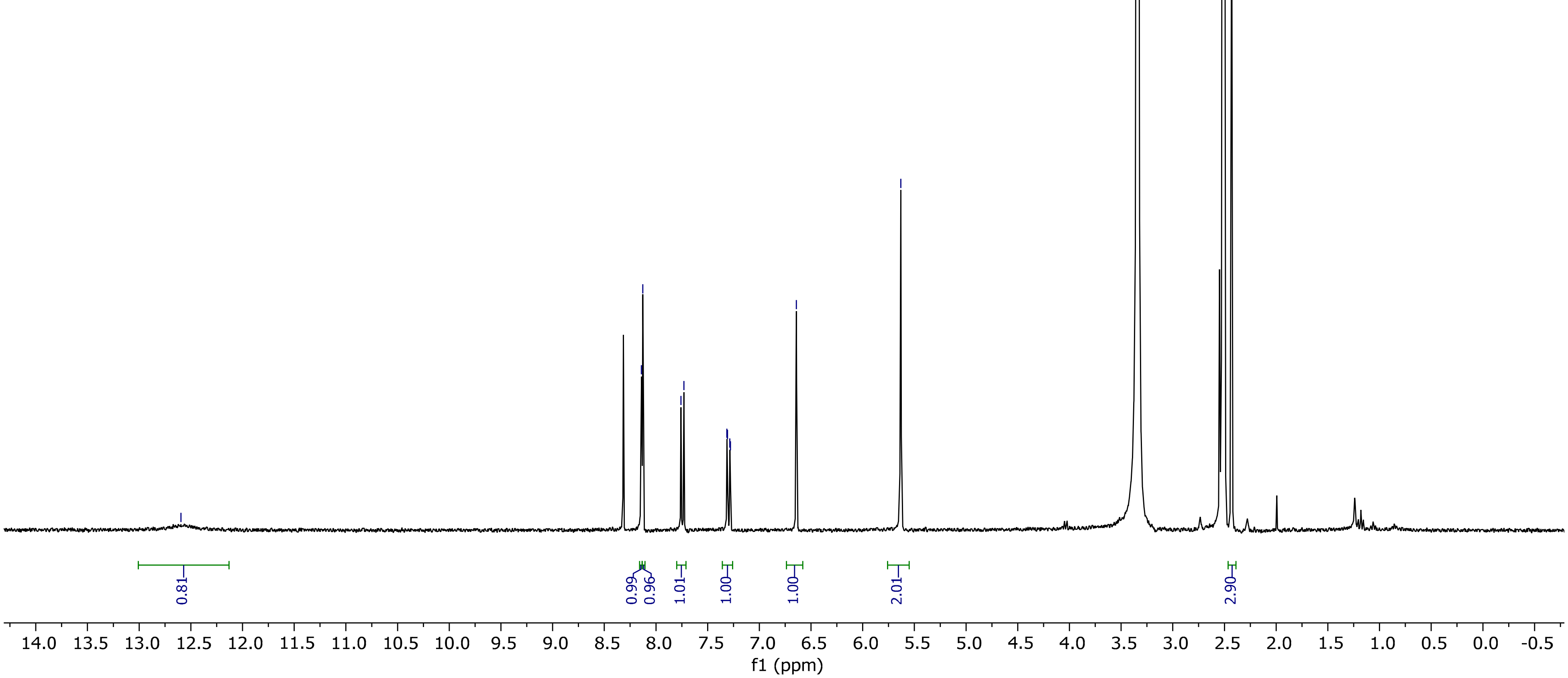

^{13}C NMR (75.5 MHz) in DMSO-d6

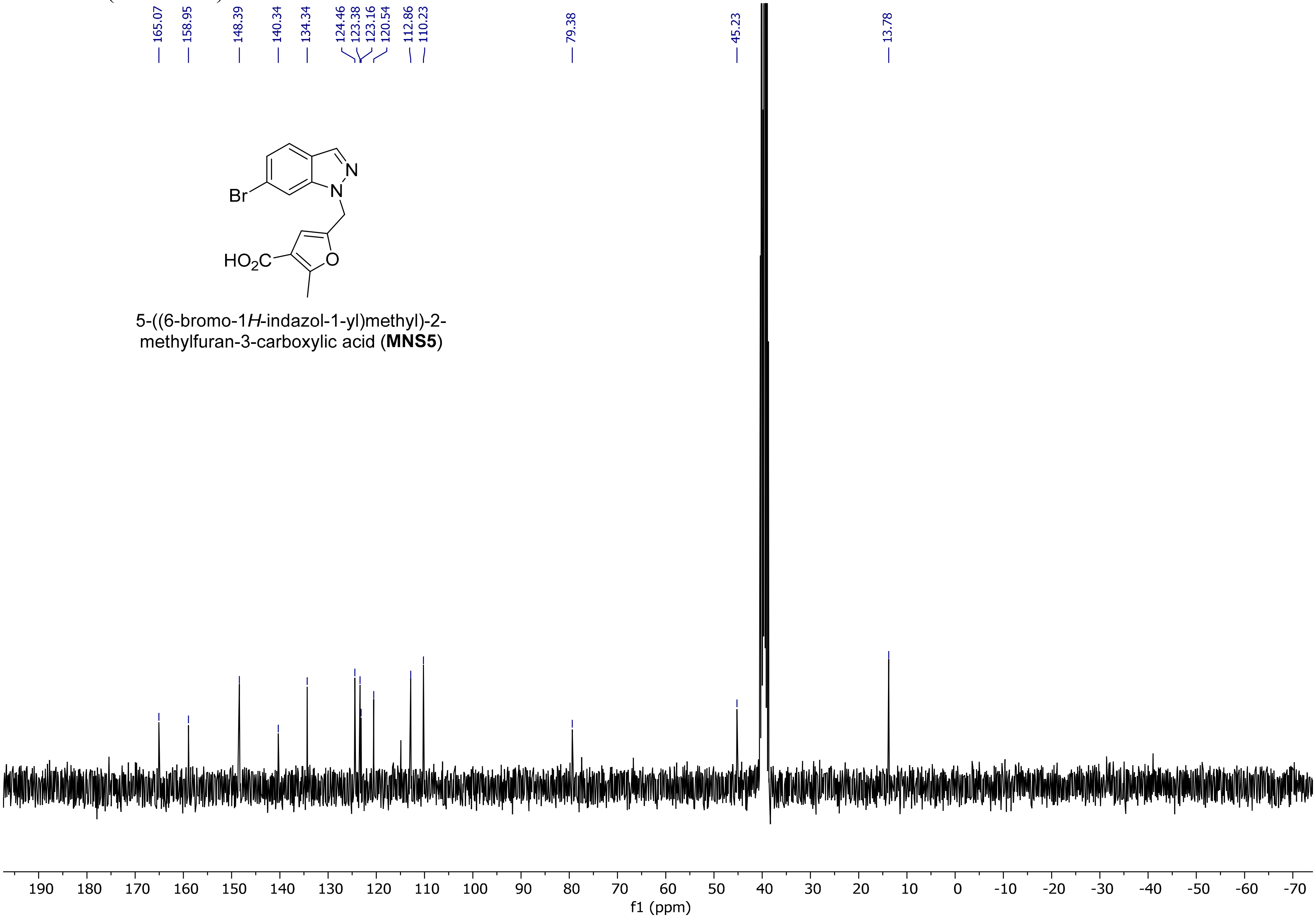

5-((6-bromo-1*H*-indazol-1-yl)methyl)-2-methylfuran-3-carboxylic acid (**MNS5**)

^{1}H NMR (300 MHz) in DMSO-d6

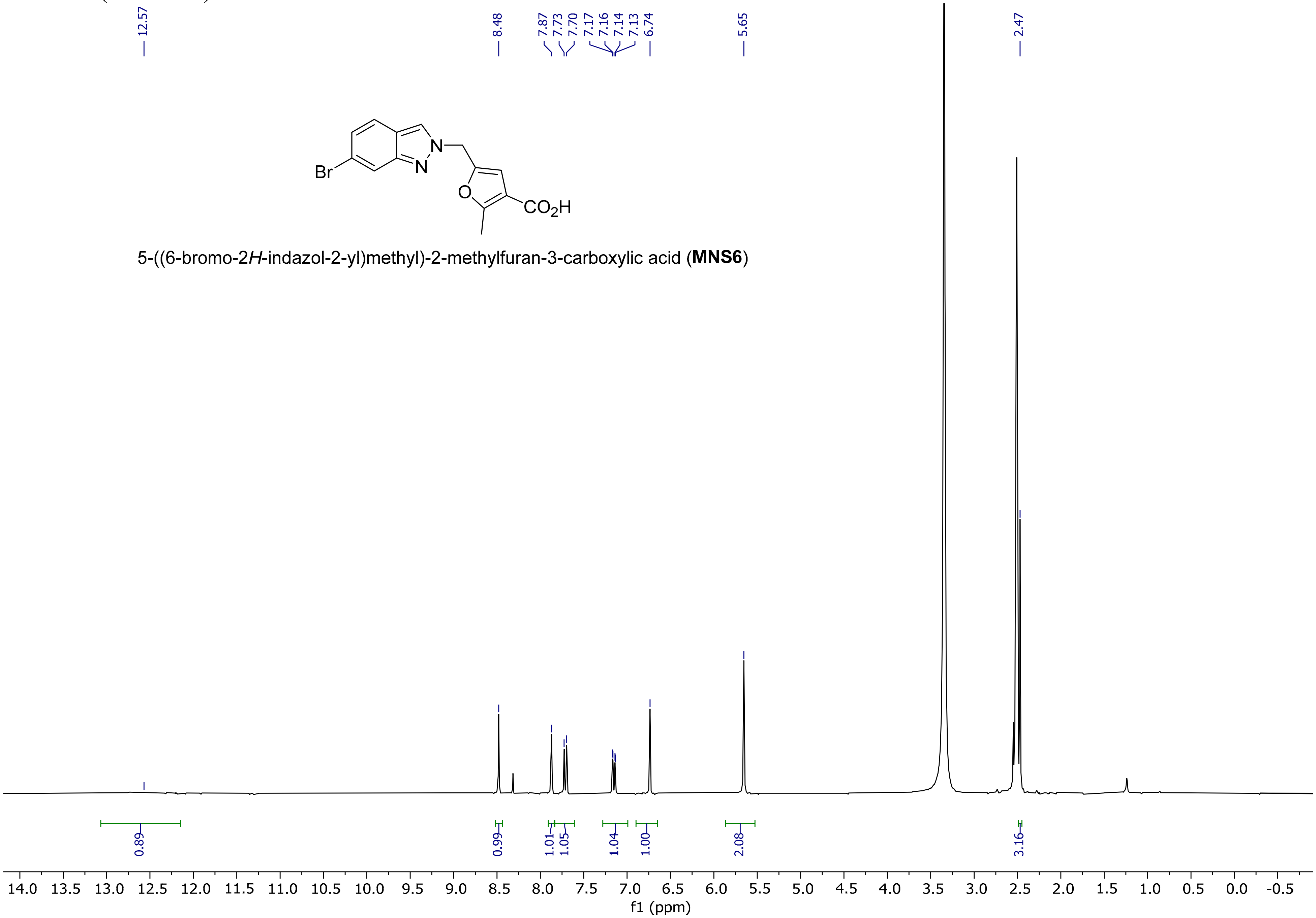

5-((6-bromo-2*H*-indazol-2-yl)methyl)-2-methylfuran-3-carboxylic acid (**MNS6**)

^{13}C NMR (75.5 MHz) in DMSO-d6

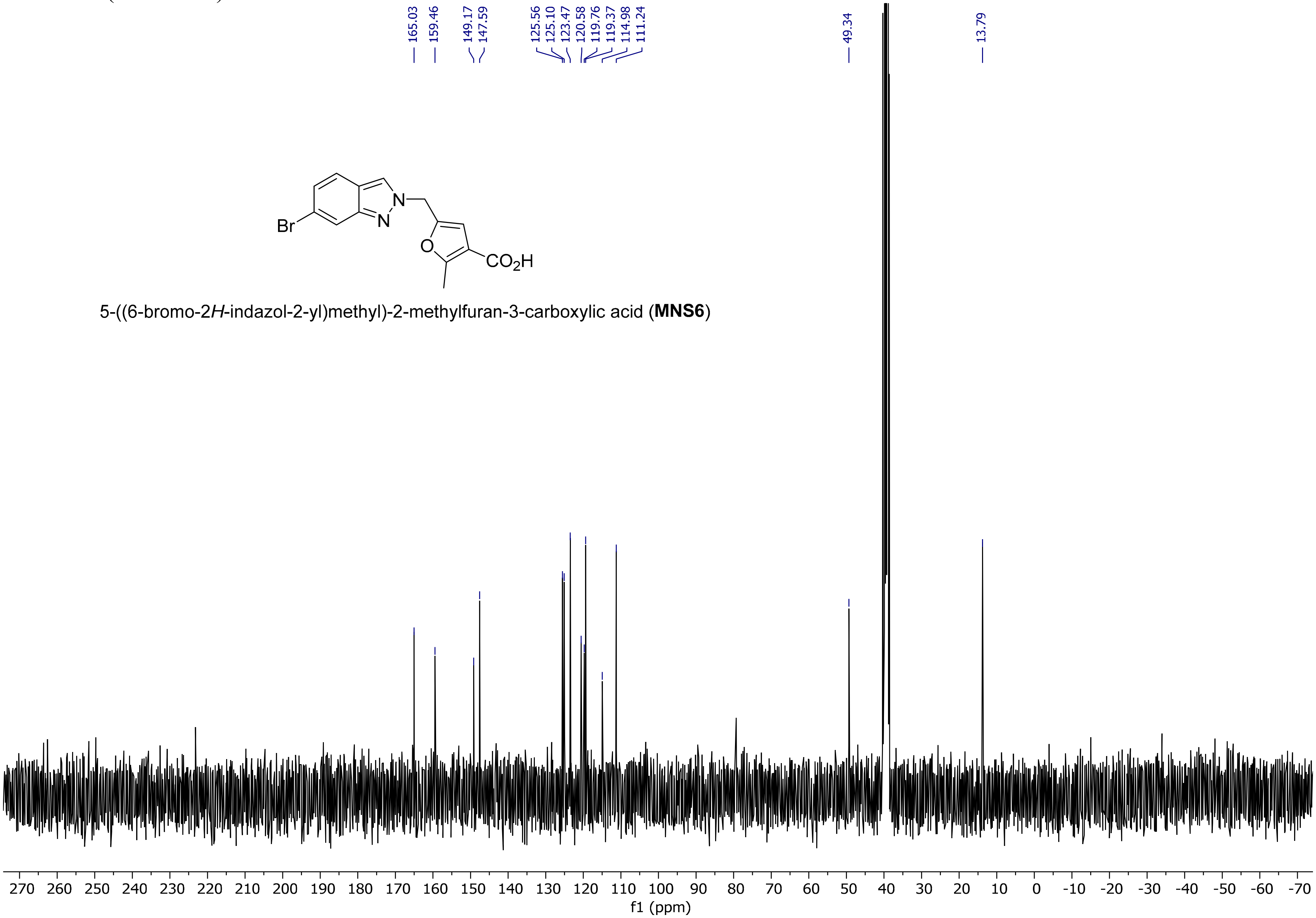

5-((6-bromo-2*H*-indazol-2-yl)methyl)-2-methylfuran-3-carboxylic acid (**MNS6**)

^{1}H NMR (300 MHz) in $CDCl_3$

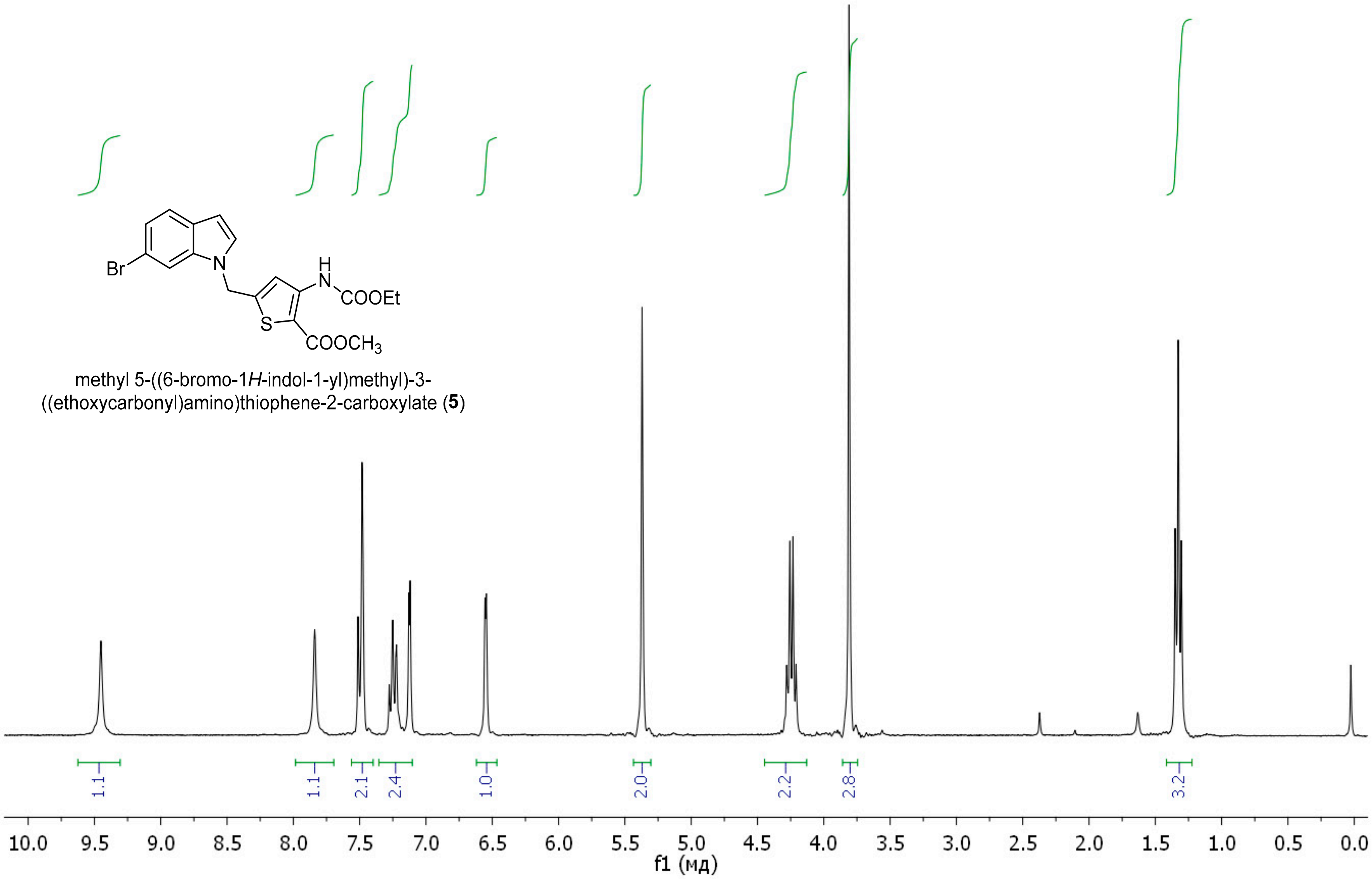

^{13}C NMR (75.5 MHz) in $CDCl_3$

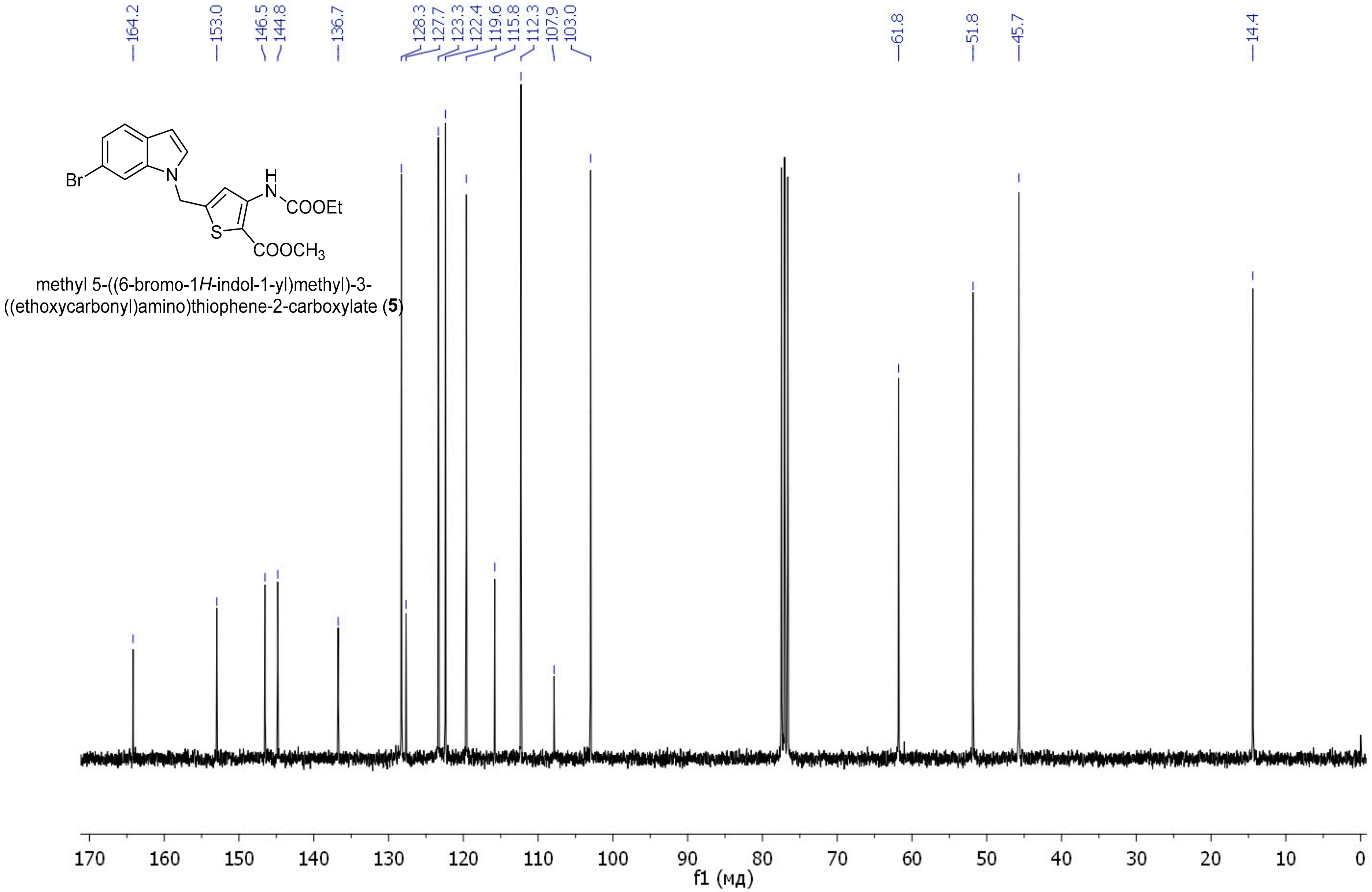

^{1}H NMR (300 MHz) in $CDCl_3$

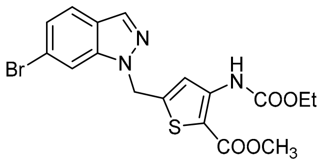

methyl 5-((6-bromo-1*H*-indazol-1-yl)methyl)-3-((ethoxycarbonyl)amino)thiophene-2-carboxylate (**7**)

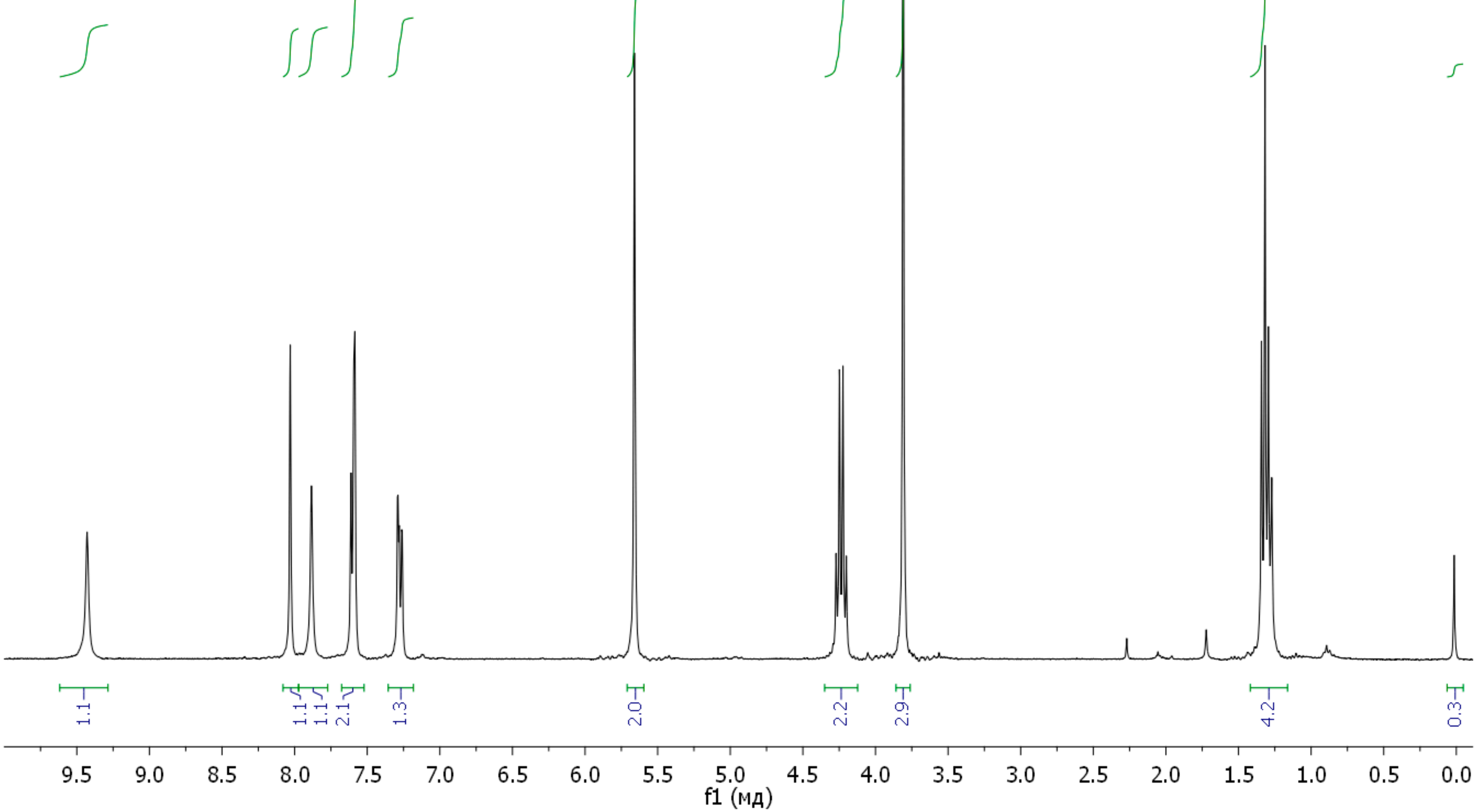

^{13}C NMR (75.5 MHz) in $CDCl_3$

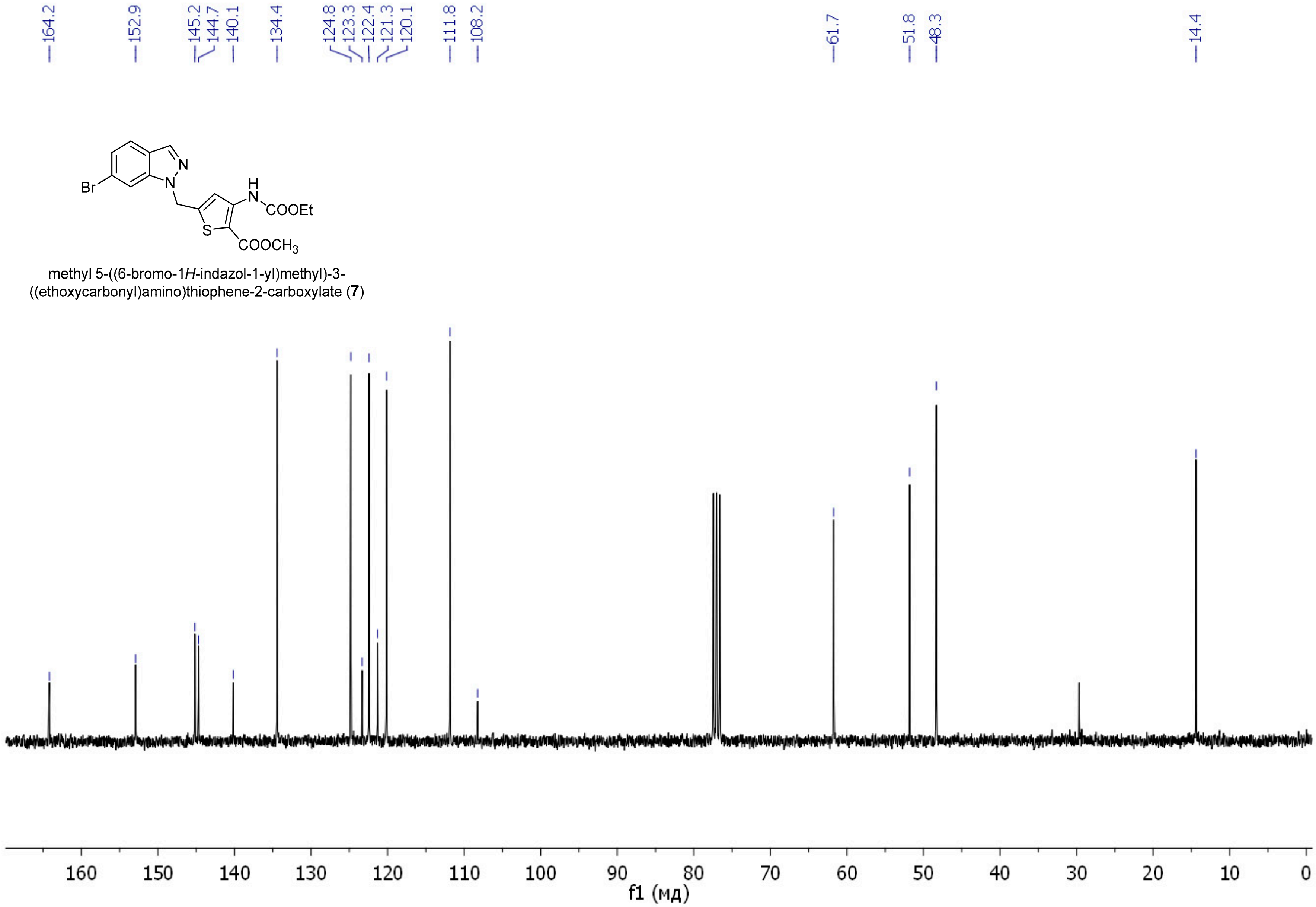

methyl 5-((6-bromo-1*H*-indazol-1-yl)methyl)-3-((ethoxycarbonyl)amino)thiophene-2-carboxylate (**7**)

^{1}H NMR (300 MHz) in $CDCl_3$

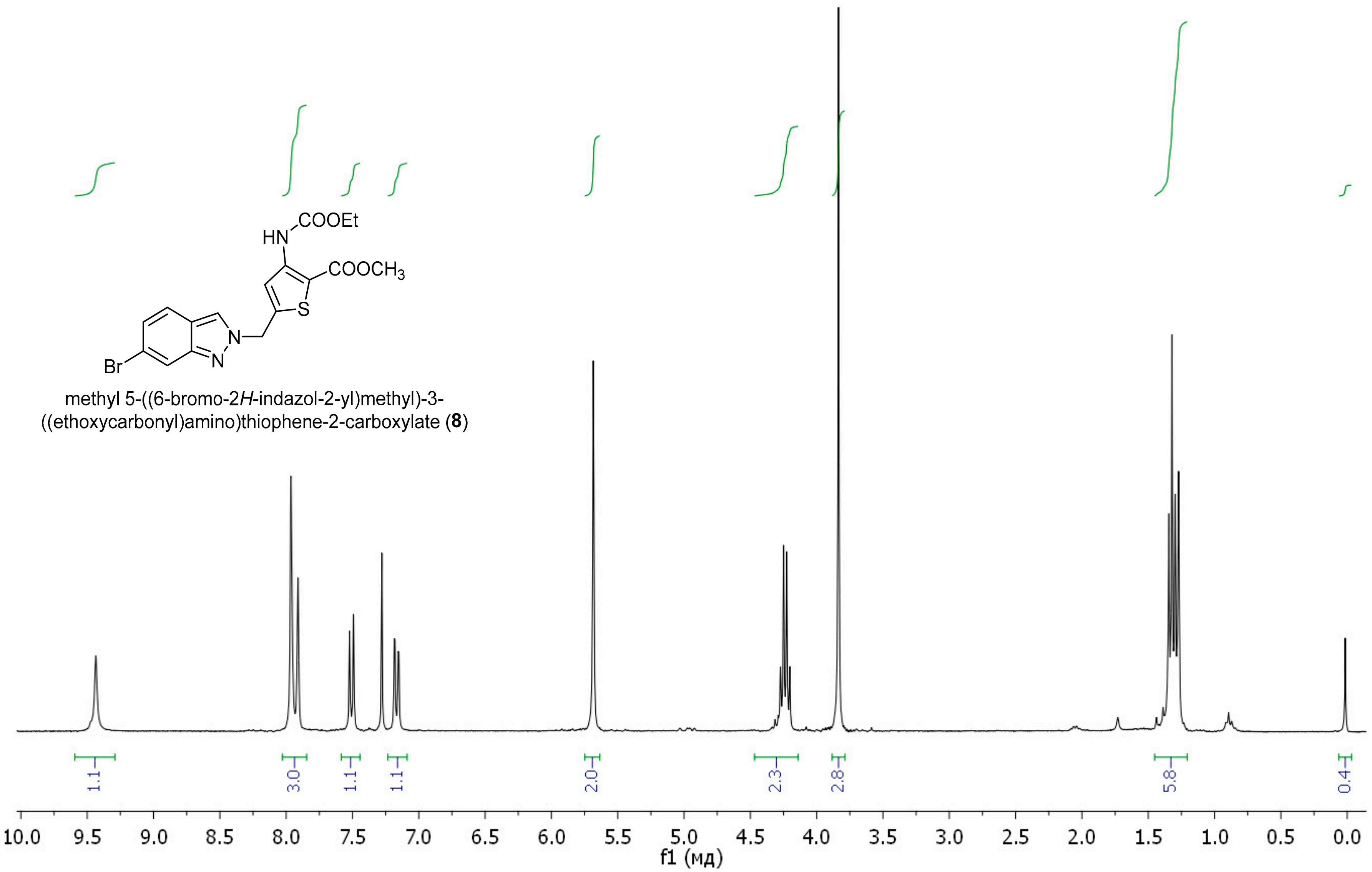

^{13}C NMR (75.5 MHz) in $CDCl_3$

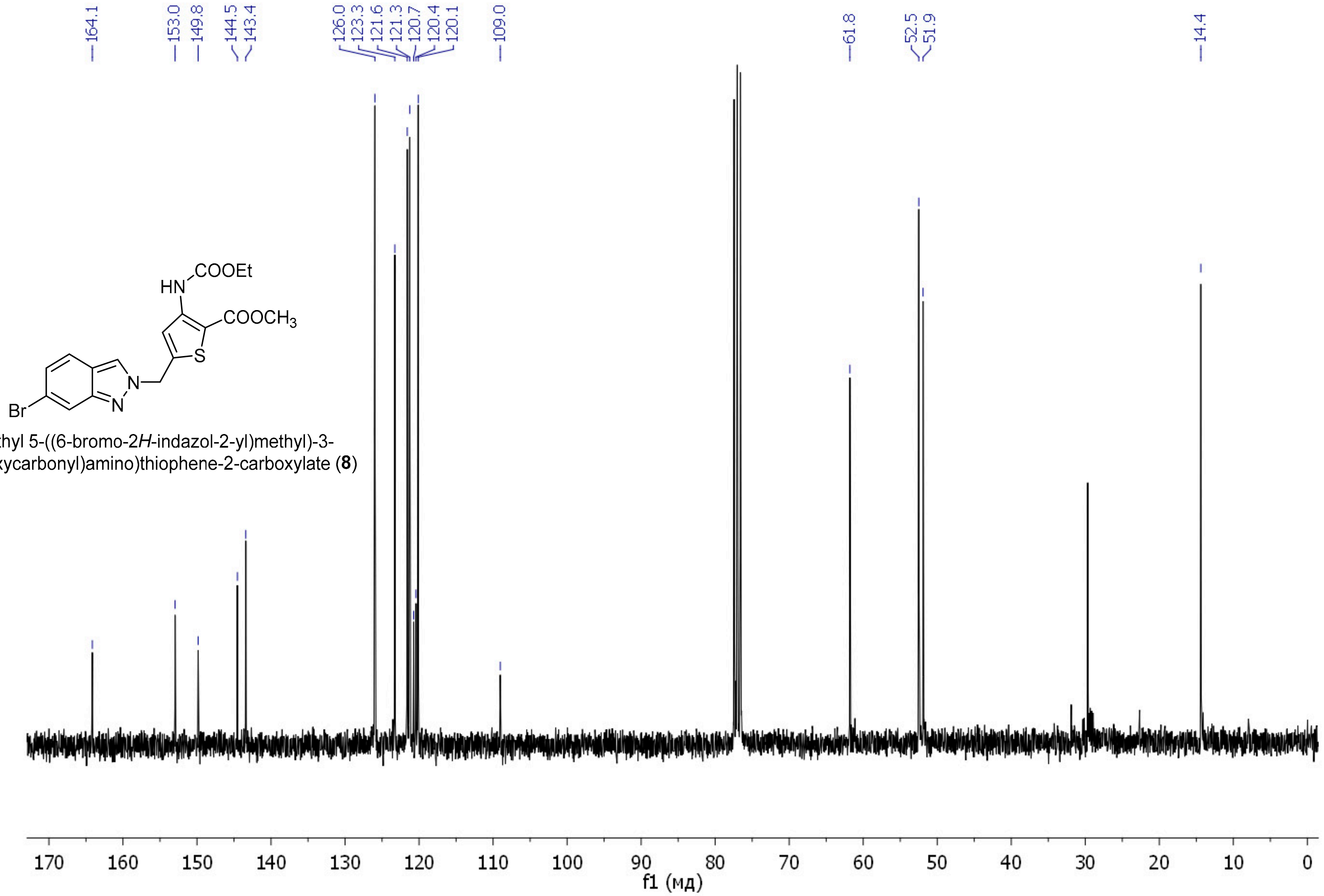

methyl 5-((6-bromo-2*H*-indazol-2-yl)methyl)-3-((ethoxycarbonyl)amino)thiophene-2-carboxylate (**8**)

^{1}H NMR (300 MHz) in D_2O

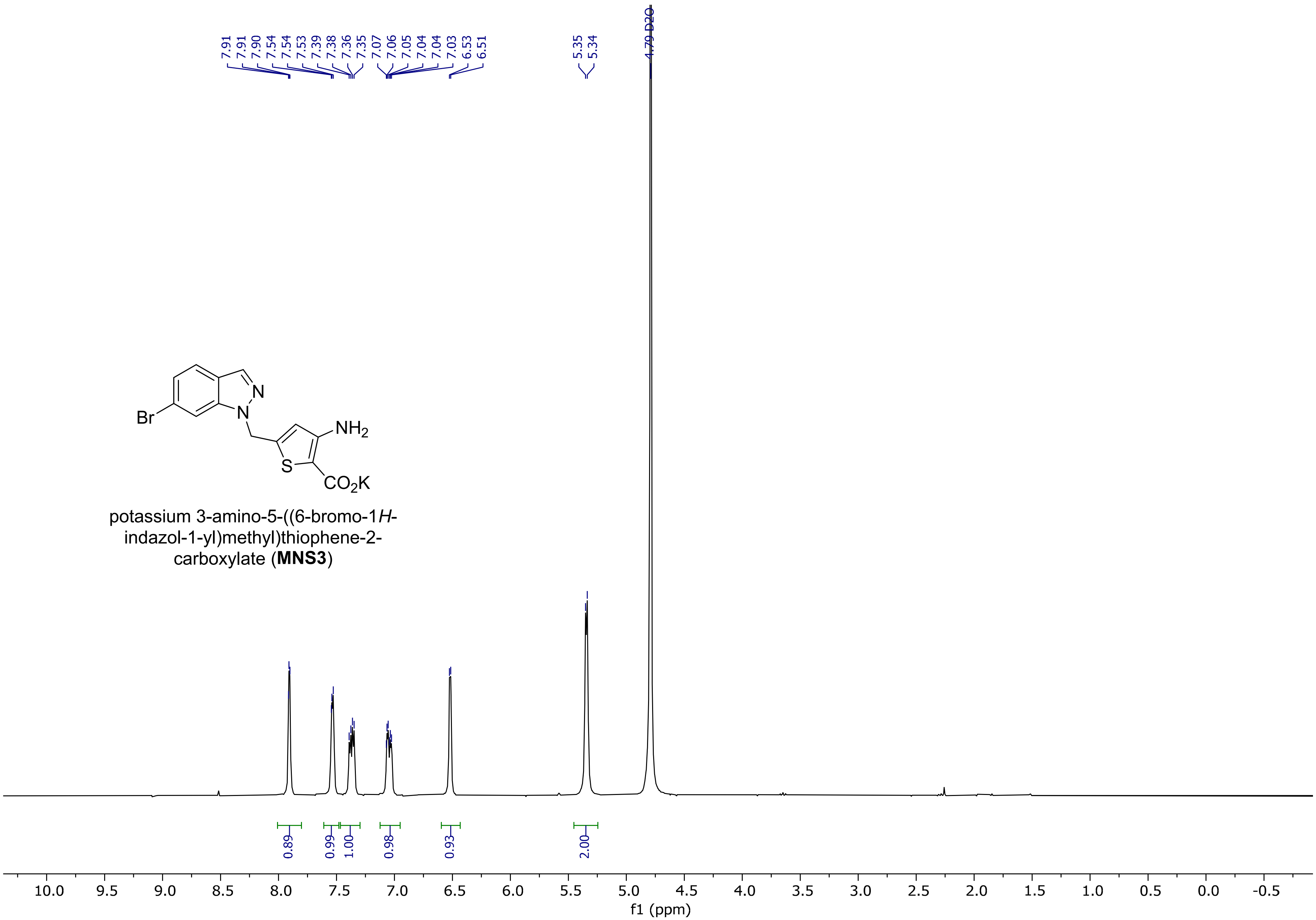

potassium 3-amino-5-((6-bromo-1*H*-indazol-1-yl)methyl)thiophene-2-carboxylate (**MNS3**)

^{13}C NMR (75.5 MHz) in D_2O

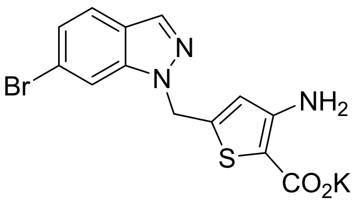

potassium 3-amino-5-((6-bromo-1*H*-indazol-1-yl)methyl)thiophene-2-carboxylate (**MNS3**)

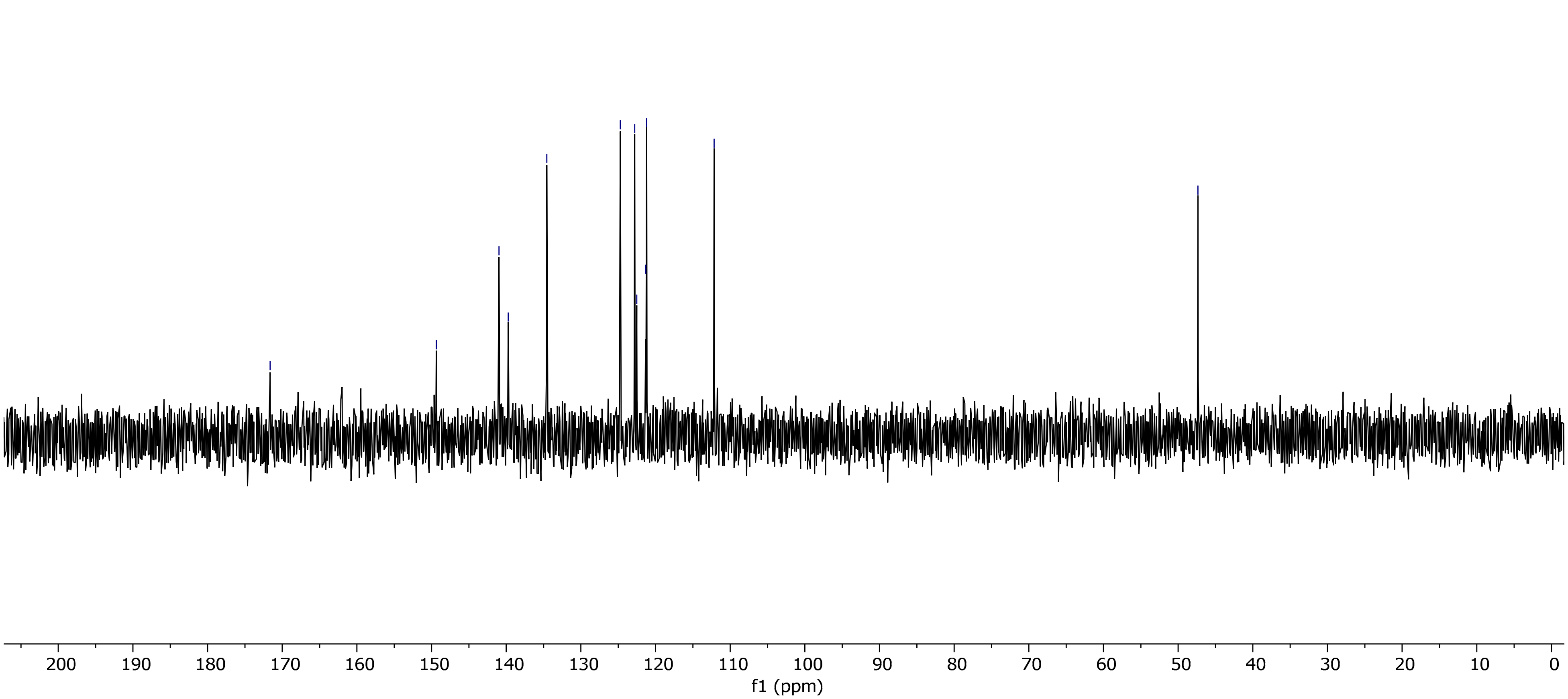

^{1}H NMR (300 MHz) in D_2O

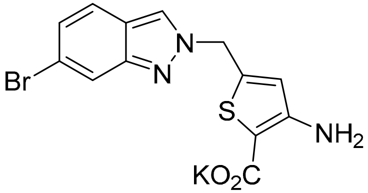

potassium 3-amino-5-((6-bromo-2*H*-indazol-2-yl)methyl)thiophene-2-carboxylate (**MNS4**)

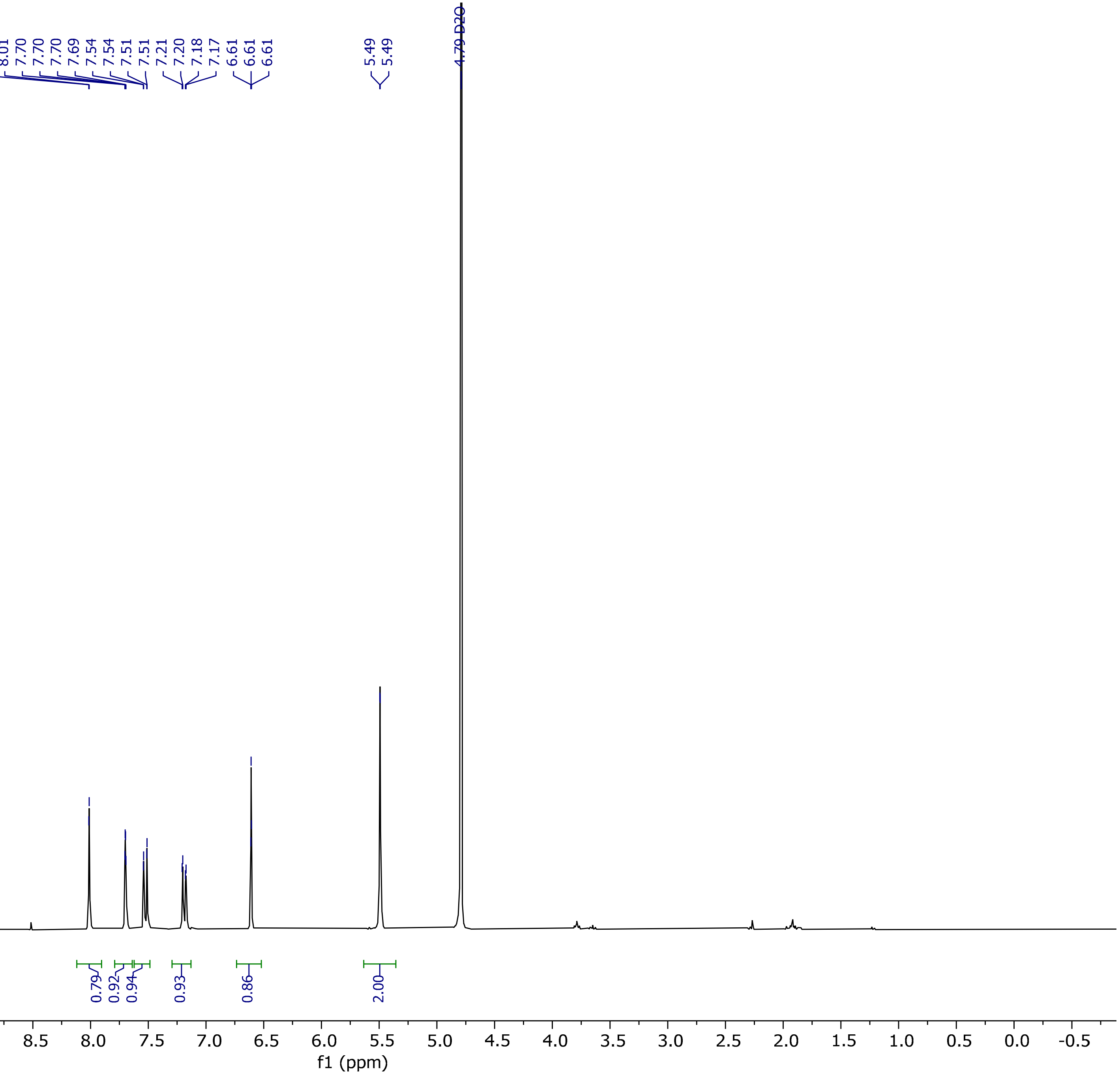

^{13}C NMR (75.5 MHz) in D_2O

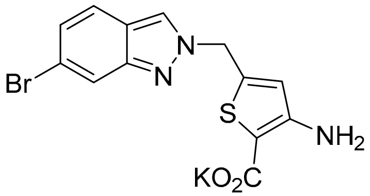

potassium 3-amino-5-((6-bromo-2*H*-indazol-2-yl)methyl)thiophene-2-carboxylate (**MNS4**)

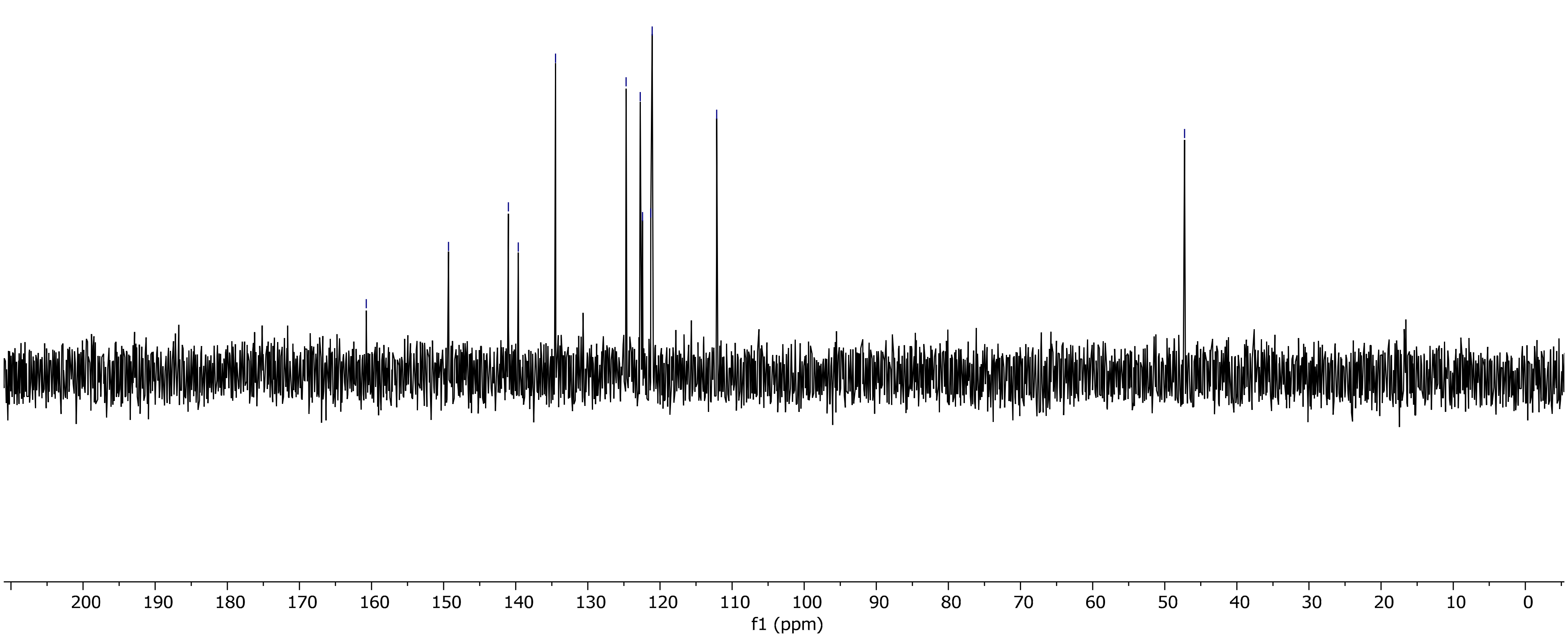

^{1}H NMR (600 MHz) in D_2O

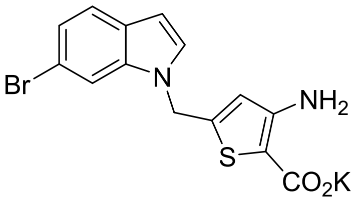

potassium 3-amino-5-((6-bromo-1*H*-indol-1-yl)methyl)thiophene-2-carboxylate (**MNS2**)

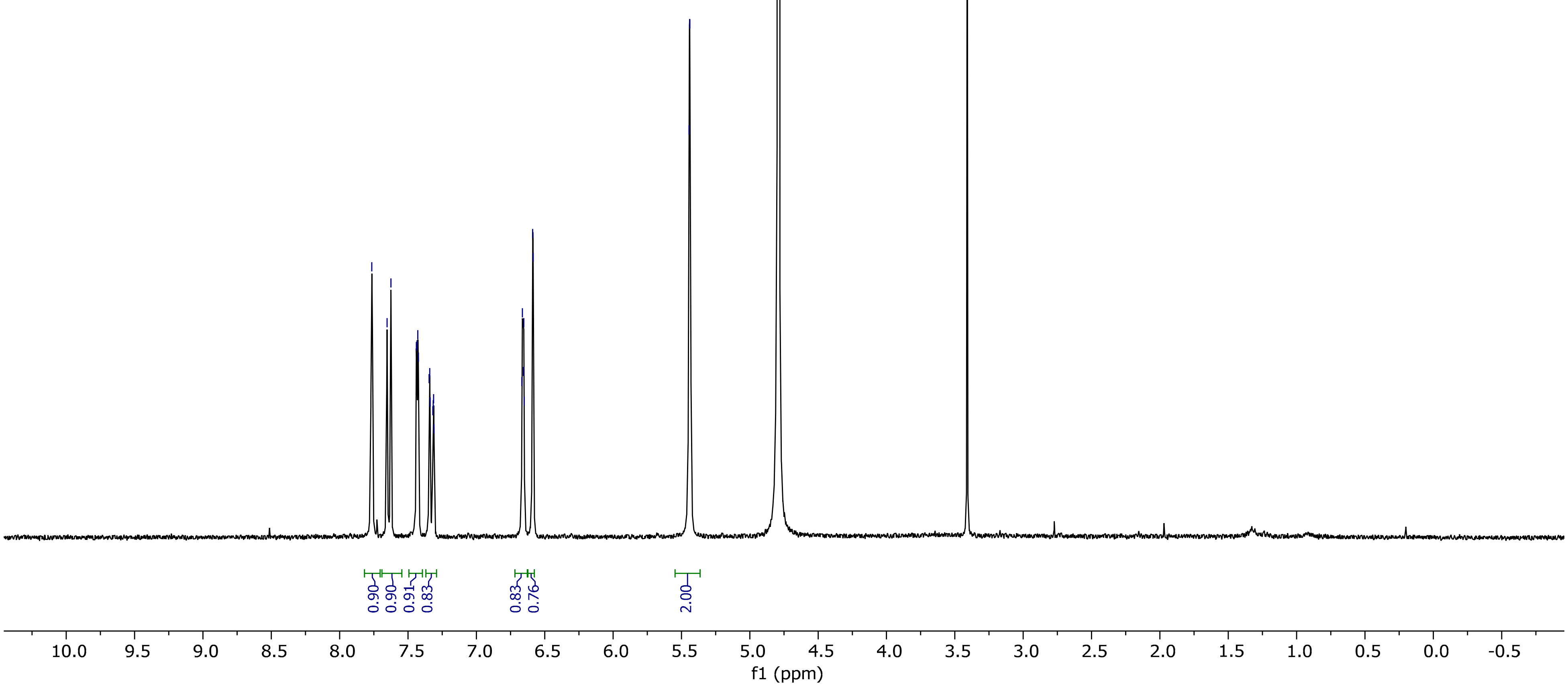

^{13}C NMR (151 MHz) in D_2O

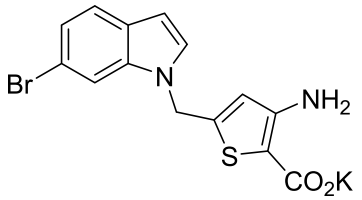

potassium 3-amino-5-((6-bromo-1*H*-indol-1-yl)methyl)thiophene-2-carboxylate (**MNS2**)

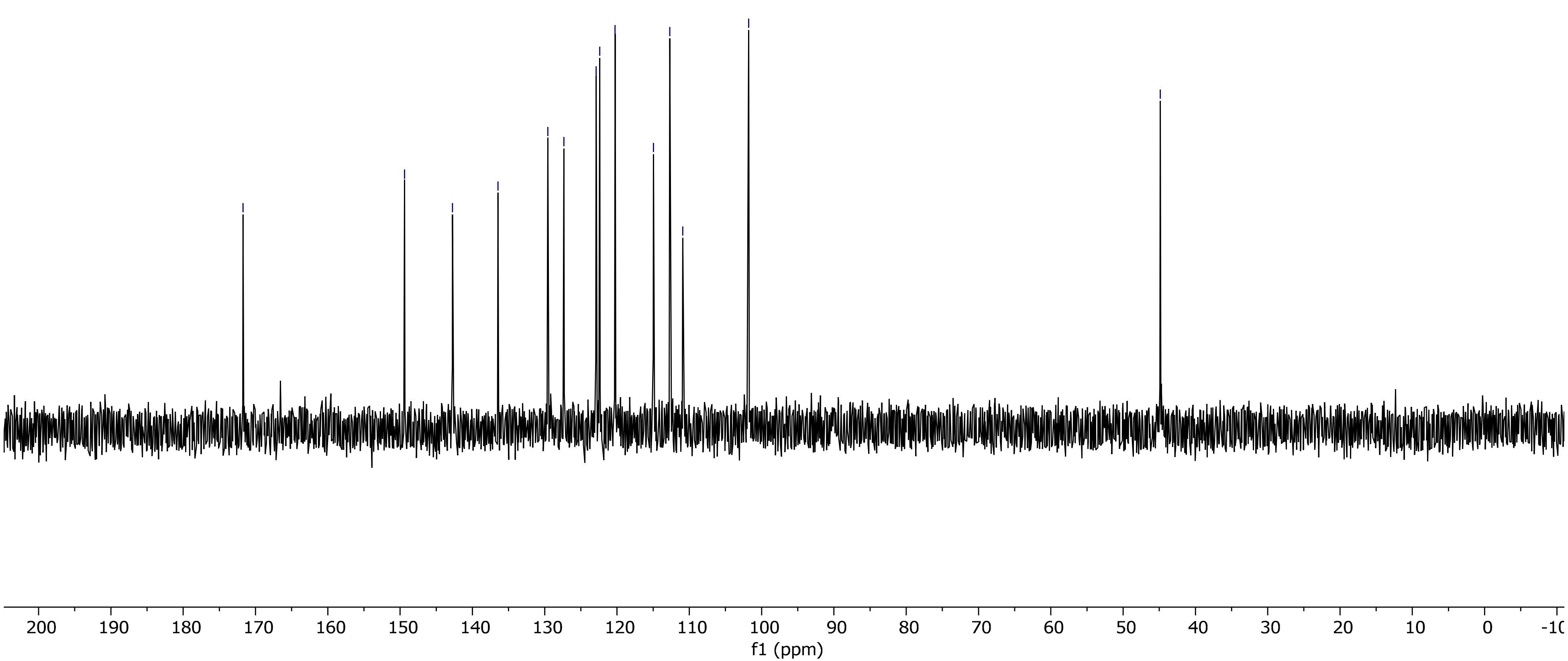

Supplement: Supplementary file 1 [file molecules-30-00388-s001.zip › molecules-3339309-supplementary.pdf]
